# Molecular and biochemical analysis of the first ARA6 homologue, a RAB5 GTPase, from green algae

**DOI:** 10.1093/jxb/ert322

**Published:** 2013-10-14

**Authors:** Marion C. Hoepflinger, Anja Geretschlaeger, Aniela Sommer, Margit Hoeftberger, Tomoaki Nishiyama, Hidetoshi Sakayama, Peter Hammerl, Raimund Tenhaken, Takashi Ueda, Ilse Foissner

**Affiliations:** ^1^Plant Physiology/Cell Biology, University of Salzburg, Hellbrunnerstrasse 34, 5020 Salzburg, Austria; ^2^Advanced Science Research Center, Kanazawa University, 13-1 Takara-machi, Kanazawa, Ishikawa 920-0934, Japan; ^3^Department of Biology, Graduate School of Science, Kobe University, 1-1 Rokkodai, Nada-ku, Kobe, Hyogo 657-8501, Japan; ^4^Central Animal Facility, University of Salzburg, Hellbrunnerstr. 34, 5020 Salzburg, Austria; ^5^Department of Biological Sciences, Graduate School of Science, The University of Tokyo, Bunkyo-ku, Tokyo 113-0033, Japan

**Keywords:** ARA6, *Chara australis*, endosomal trafficking, multivesicular endosome, plant-specific RAB5 GTPase, plasma membrane, *trans*-Golgi network.

## Abstract

RAB5 GTPases are important regulators of endosomal membrane traffic in yeast, plants, and animals. A specific subgroup of this family, the ARA6 group, has been described in land plants including bryophytes, lycophytes, and flowering plants. Here, we report on the isolation of an *ARA6* homologue in a green alga. *CaARA6* (*CaRABF1*) from *Chara australis*, a member of the Characeae that is a close relative of land plants, encodes a polypeptide of 237 aa with a calculated molecular mass of 25.4kDa, which is highly similar to *ARA6* members from *Arabidopsis thaliana* and other land plants and has GTPase activity. When expressed in *Nicotiana benthamiana* leaf epidermal cells, fluorescently tagged CaARA6 labelled organelles with diameters between 0.2 and 1.2 µm, which co-localized with fluorescently tagged AtARA6 known to be present on multivesicular endosomes. Mutations in the membrane-anchoring and GTP-binding sites altered the localization of CaARA6 comparable to that of *A. thaliana* ARA6 (RABF1). In characean internodal cells, confocal immunofluorescence and immunogold electron microscopy with antibodies against AtARA6 and CaARA6 revealed ARA6 epitopes not only at multivesicular endosomes but also at the plasma membrane, including convoluted domains (charasomes), and at the *trans*-Golgi network. Our findings demonstrate that ARA6-like proteins have a more ancient origin than previously thought. They indicate further that ARA6-like proteins could have different functions in spite of the high similarity between characean algae and flowering plants.

## Introduction

The eukaryotic cell is characterized by membrane-bound compartments that exchange material by membrane trafficking mediated by vesicles or tubules. Membrane trafficking includes exo- and endocytosis during which intermediate carriers fuse with or detach from the plasma membrane. In plant sciences, exocytosis of cell-wall material has been a focus of plant cell research for decades (Richter *et al.*, 2009). Endocytosis had long been doubted because of the cell turgor pressure (see references in Aniento and Robinson, 2005; Murphy *et al.*, 2005). This changed rapidly with the introduction of FM dyes and filipin as endocytic tracers and with the development of new molecular biological techniques (e.g. Meckel *et al.*, 2004). On recent years, it has become clear that endocytosis of plasma-membrane proteins and extracellular components is essential for establishing plant morphology and adaption to environmental cues (Reyes *et al.*, 2011; Contento and Bassham, 2012; Park and Jürgens, 2012).

Membrane trafficking must be strictly regulated to ensure proper targeting. Among the key regulators are RAB GTPases, a family of small GTPases with a molecular mass of 20–25kDa (Schwartz *et al.*, 2007), which cycle between GTP and GDP conformations. In their activated, GTP-bound state, RAB GTPases localize at membranes, where they recruit effector molecules and promote downstream reactions including tethering of transport vesicles or organelles to target membranes required for docking and fusion. Hydrolysis of GTP causes the release of RAB GTPases into the cytosol in a RAB GDP dissociation inhibitor-dependent manner. Activation and inactivation of GTPases are regulated by guanine nucleotide exchange factors and by GTPase activating proteins; these regulators drive the GTPase cycle of RAB proteins (Goh *et al.*, 2007; Saito and Ueda, 2009).

Among RAB GTPases, members of the RAB5 family are responsible for endosomal trafficking in yeast, animal, and plant cells (Schwartz *et al.*, 2007). In *Arabidopsis thaliana*, the RAB5 group consists of RHA1 (RABF2a), ARA7 (RABF2b), and ARA6 (RABF1) (Ueda *et al.*, 2001, 2004). ARA7 and RHA1 have a similar structure to mammalian RAB5 GTPases and are therefore considered to play similar roles. ARA7 and RHA1 are involved in endocytosis and transport towards the vacuole in a consistent manner (e.g. Sohn *et al.*, 2003; Kotzer *et al.*, 2004; Dhonukshe *et al.*, 2006). ARA6, however, was found to be plant specific and differs from conventional RAB members in the sequence responsible for membrane anchoring (N-terminal myristoylation and palmitoylation instead of C-terminal prenylation; Ueda *et al.*, 2001).

The localization and function of ARA6 are not completely understood yet, while recent studies have been highlighting its unique properties. In electron microscopy (EM) studies, AtARA6 has been shown to localize at multivesicular endosomes (MVEs; Haas *et al.*, 2007), like ARA7 with which it considerably overlaps. This co-localization was confirmed by using the phosphatidylinositol 3-monophosphate kinase inhibitor wortmannin (WM), which causes the enlargement of MVEs due to homotypic fusion (Wang *et al.*, 2009). Co-localization of AtARA6-positive organelles with the endocytic tracer FM4-64 (Ueda *et al.*, 2001; Uemura *et al.*, 2004), the sterol-binding probe filipin III (Grebe *et al.*, 2003), and the boron transporter BOR1 (Ebine *et al*., 2011) further indicates a role of ARA6 in endosomal trafficking. Recent studies indicate that ARA6 also localizes to the plasma membrane, where it plays a regulatory role in the formation of a SNARE complex containing endosome-associated VAMP727 and plasma membrane-localizing SYP121 (Ebine *et al*., 2011). A mutated form of ARA6 has been also shown to perturb vacuolar trafficking of soluble cargos in tobacco cells (Bottanelli *et al.*, 2011). These results imply distinctive functions of ARA6 in different endosomal trafficking pathways, while molecular details of the apparent dual function remain unclear.

So far, *ARA6* homologues have been identified in the genomes of angiosperms, the lycophyte *Selaginella moellendorffii* and the bryophyte *Physcomitrella patens.* Only conventional RAB5 members were found in green algae. ARA6 was therefore reported to be specific to land plants (Ebine and Ueda, 2009). In this study, we investigated *Chara australis*, a member of the characean green algae. The morphological, biochemical, and molecular data of the Characeae strongly suggest that they are close relatives of land plants (Graham *et al.*, 2000; Karol *et al.*, 2001; McCourt *et al.*, 2004; Tanabe *et al.*, 2005; Nishiyama, 2007; Turmel *et al.*, 2007). However, the phylogenetic placement of the Charalean algae within the streptophytes (land plants and charophycean green algae sensu Mattox and Stewart (1984) is still controversial (Finet *et al.*, 2010; Wodniok *et al.*, 2011; Laurin-Lemay *et al.*, 2012; Timme *et al.*, 2012). Their multicellular thallus (Supplementary Fig. S1A at *JXB* online) consists of regularly alternating groups of small nodal cells and cylindrical internodes, which may attain a length of up to several centimetres. They have a characteristic cytoarchitecture consisting of a cortical cytoplasm that includes files of stationary chloroplasts and a streaming endoplasm harbouring up to several thousand nuclei, Golgi bodies, *trans*-Golgi networks (TGNs), and MVEs (Supplementary Fig. S1B). Cytoplasmic streaming is generated by interaction of myosin-coated organelles and actin filament bundles attached to the inner side of the chloroplast files. Because of rapid cytoplasmic streaming, huge cell size, and simple geometry, internodal cells have been used as a model for studying various aspects of plant cell biology for a long time (Tazawa and Shimmen, 2001; Braun *et al.*, 2007), including endo- and exocytosis in control and wounded cells (Klima and Foissner, 2011; Foissner and Wasteneys, 2012). The plasma membranes in internodal cells of the genus *Chara* show an interesting detail: they consist of smooth and convoluted regions, called charasomes. These charasomes are involved in generation of acid bands at the cell surface, which are required for efficient carbon uptake necessary for photosynthesis (Schmölzer *et al.*, 2011, and references therein).

During the course of this study, we identified an ARA6-like protein in the green alga *C. australis*. The high sequence similarity to ARA6 from flowering plants, lycophytes, and mosses in addition to the location of CaARA6 (CaRABF1) at the plasma membrane, the TGN, and MVEs suggested its involvement in endosomal trafficking. Our data indicate the more ancient origin of this plant-specific RAB5 GTPase required for the plant-unique trafficking pathway.

## Materials and methods

### Algal material and culture conditions

For this study, we mainly investigated male thalli of *C. australis* R.Br., which is widely used for cell biological research. In our earlier papers, we referred to this alga as *Chara corallina* according to the monograph of Characeae by Wood and Imahori (1965) who amalgamated both species. The fact that *C. australis* is dioecious and possesses only the half number of chromosomes (14) compared with the monoecious *C. corallina* (≥28), however, justifies the retainment of a separate species (Casanova, 2005). The internodal cells of *C. australis* are too large for high-pressure freezing. For EM, we therefore used the smaller internodal cells of *Chara braunii* Gm. Recent molecular phylogenetic studies demonstrated a close relationship between *C. braunii* and *C. australis* (Sakayama *et al.*, 2009; Kato *et al.*, 2010).

Thalli of *C. australis* and *C. braunii* were grown in a substrate of soil, peat, and sand in 10–50 l aquaria filled with distilled water. The temperature was about 20 °C and fluorescent lamps provided a 16/8h light/dark cycle. Non-elongating, mature internodal cells of the branchlets were harvested 1 d prior to experiments, trimmed of neighbouring internodal cells, and left overnight in artificial fresh water (10^–3^ M NaCl, 10^–4^ M KCl, 10^–4^ M CaCl_2_).

### 454 sequencing of *C. australis*

Thalli of *C. australis* were collected from cultures as described above, rinsed with distilled water, gently blotted dry, frozen in liquid nitrogen, and homogenized using a mortar and pestle. Total RNA was extracted with TRI Reagent according to manufacturer’s instructions (Sigma-Aldrich, Schnelldorf, Germany). Transcriptomic data of *C. australis* were produced from a normalized random-primed cDNA library followed by 454 sequencing (Roche GS FLX system; Eurofins MWG, Ebersberg, Germany). BLAST analyses (Altschul *et al.*, 1990) were performed using annotated ARA6 sequences to reveal putative *C. australis ARA6* cDNA. The accession number for CaARA6 is HF968767.

### Cloning of an *ARA6* homologue from *C. australis*

Total RNA was extracted from whole *C. australis*. RNA was prepared as described above. Residual DNA was removed by treatment with RNase-free DNase (Fermentas, St Leon-Rot, Germany). First-strand cDNA synthesis was performed with 1 µg of total RNA using Moloney murine leukemia virus reverse transcriptase (RevertAid; Fermentas) and an anchored oligo(d)T primer-mix according to the supplier’s protocol.

For cloning of the *ARA6*-like gene from *C. australis*, the following primers with restriction sites were designed: ARA6-Fwd-1 with *Bam*HI and ARA6-Rev-1 with *Hin*dIII (for primer sequences, see Supplementary Table S1 at *JXB* online), based on the cDNA sequence of *Chara*. PCR was performed with Phusion High-Fidelity DNA polymerase (Thermo Scientific, Vienna, Austria) using single-stranded cDNA as the template. The amplified cDNA sequence was digested with *Bam*HI and *Hin*dIII and cloned into a pQE30 *Escherichia coli* (Qiagen, Hilden, Germany) expression vector.

### Phylogenetic analysis

Phylogenetic analysis was performed with the neighbour-joining method (Saitou and Nei, 1987), using the distance under JTT model (Jones *et al.*, 1992). The procedure was essentially as described by Banks *et al.* (2011), but the bootstrap (Felsenstein, 1985) was increased to 1000 replicates. CaARA6 was used to query the nr database with BLAST (Altschul *et al.*, 1997), and up to 700 genes were recovered and aligned with MAFFT v.6.811b using the einsi setting (Katoh *et al.*, 2005). Unambiguously aligned sites were selected, and genes that lacked part of the conserved region were removed using MacClade v.4.08 (Maddison and Maddison, 2000). Distance calculation and tree reconstruction were performed with PHYLIP v.3.65 (Felsenstein, 2005). Trees were reconstructed iteratively, reducing the terminal taxa. At this stage, the taxa were chosen such that a large part of the tree was retained but closely related genes were reduced.

### Expression and purification of recombinant CaARA6

The pQE30-CaARA6 construct was transformed into the XL-1 strain of *E. coli* (Stratagene, La Jolla, USA) and cells were routinely grown in LB medium (250ml of medium in a 1 l flask) containing 100 µg ml^–1^ of ampicillin at 37 °C to OD_600_ between 0.6 and 1.0 under vigorous shaking. Cultures were cooled to 20 °C and protein expression was induced by addition of 0.5mM isopropyl 1-thio-β-d-galactopyranoside (IPTG). After 20h vigorous shaking at 20 °C, cells were cooled to 4 °C for 30min before harvesting.

All the following purification steps were performed at 4 °C. The culture was centrifuged and the pellet was resuspended in 3ml g^–1^ of pellet of equilibration buffer containing 50mM NaH_2_PO_4_, 300mM NaCl and 20% glycerol, having a pH of 8.0 adjusted with NaOH. Lysozyme was added at a final concentration of 1mg ml^–1^ and the culture was shaken on ice for 30min. After incubation, the suspension was sonicated and remaining insoluble residues were removed by centrifugation for 5min at 13000*g*. For purification of His-tagged recombinant CaARA6, prepacked Protino^®^Ni-TED 1000 columns (Macherey-Nagel) were used according to manufacturer’s instructions. Desalting of eluted protein was performed using an Amicon centrifugal filter unit (Millipore, Billerica, USA; molecular mass cut-off of 10kDa) against buffer containing 50mM HEPES/KOH (pH 7.5), 2mM MgCl_2_, and 300mM NaCl. After addition of 20% glycerol, recombinant protein was stored at –20 °C and analysed by SDS-PAGE and Western blotting.

### GTPase activity

The GTPase activity of CaARA6 was tested with a Transcreener^®^ GDP FI assay (BellBrookLabs, Madison, WI, USA) using purified recombinant protein. This highly sensitive assay is based on the detection of GDP, which displaces fluorescently labelled GDP–Alexa Fluor 594 tracer bound to a GDP antibody–IR dye quencher conjugate, hence setting free the fluorescent tracer, which can be detected and quantified.

Recombinant purified ARA6 proteins were diluted in buffer containing 50mM HEPES (pH 7.5), 4mM MgCl_2_, 2mM EGTA, 1% DMSO, and 0.01% Triton X-100. All measurements were performed in four replicates at room temperature according to the manufacturer’s instructions. The fluorescence intensity readings were performed in a 96-well plate with a Tecan Infinite^®^ M200 Pro Instrument (Tecan Austria GmbH, Grödig, Austria). A standard curve for 10 µM nucleotide and 21 µg ml^–1^ of GDP antibody–IR dye quencher conjugate was constructed and used to convert fluorescence data to GDP concentration values. Controls included (i) measurements without enzyme in the reaction mixture, and (ii) measurements with enzyme but without added nucleotide in order to determine the amount of ARA6-bound GDP, as it is frequently reported that most RAB GTPases are purified in the GDP-bound form when expressed recombinantly (e.g. Fukuda *et al.*, 2013).

### Vector construction for localization studies

For construction of ARA6 expression vectors, pGreenI-0029 (pGI; http://www.pgreen.ac.uk) was used. All PCRs were performed with Phusion High-Fidelity DNA polymerase (Thermo Scientific) according to the manufacturer’s instructions. Primer sequences are shown in Supplementary Table S1. The *A. thaliana* ubiquitin-10 promoter (*AtUBQ10p*; At4g05320) was amplified using UBQ10_fwd and UBQ10_rev and *A. thaliana* gDNA as template, which was prepared by the cetyltrimethylammonium bromide method according to Weigel and Glazebrook (2002). The promoter was cloned into pGI using *Kpn*I and *Xho*I. *A. thaliana* and *C. australis* cDNA were prepared as described above and used as template for amplification of *ARA6* using the following primers: AtARA6: AtARA6_HindIII_fwd and AtARA6_SmaI_rev; CaARA6: CaARA6_HindIII_fwd and CaARA6_SmaI_rev. Amplicons were cloned into pGI downstream of *AtUBQ10p* using the restriction enzymes *Hin*dIII and *Sma*I. For construction of C-terminal fusion proteins, either *mGFP6* (from pMDC107; see Curtis and Grossniklaus, 2003) or *mCherry* (from pCD3-960; see Nelson *et al.*, 2007) (both obtained from NASC European Arabidopsis Stock Centre) were amplified (primers: *mGFP6*: GFP6_SmaI_fwd and GFP_NotI_rev; *mCherry*: mCherry_SmaI_fwd, mCherry_NotI_rev, respectively) and cloned downstream of *ARA6* (restriction sites: *Sma*I, *Not*I). The following plasmids were obtained: pGI-*AtUBQ10p::AtARA6::GFP*, pGI-*AtUBQ10p::AtARA6::mCherry*, pGI-*AtUBQ10p::CaARA6::GFP*, and pGI-*AtUBQ10p::CaARA6::mCherry*.

CaARA6 point-mutation-carrying vectors were constructed via site-directed mutagenesis using pGI-*AtUBQ10p::CaARA6::GFP* as template vector. PCRs were performed using PfuUltra^TM^ High-Fidelity DNA polymerase (Stratagene, La Jolla, USA) and the following primers: CaAra6_C3S_fwd and CaAra6_C3S_rev; CaAra6_G2A_C3S_fwd and CaAra6_G2A_C3S_rev; CaAra6_S73N_fwd and CaAra6_S73N_rev; CaAra6_Q118L_fwd and CaAra6_Q118L_rev; and CaAra6_N172I_fwd and CaAra6_N172I_rev (Supplementary Table S1). PCR was performed as follows: 95 °C for 30 s, followed by16 cycles of 95 °C for 30 s, 55 °C for 1min, and 68 °C for 7min, followed by incubation on ice for 2min and template vector restriction using 10U of *Dpn*I (Fermentas) for 1h at 37 °C. Remaining mutated vector was transformed into *E. coli* (strain XL-1) and tested for correct mutagenesis by sequencing.

### Monoclonal antibody production

A monoclonal antibody against CaARA6 was produced by standard hybridoma techniques. Briefly, NMRI mice were immunized by intradermal injection of 10 µg of CaARA6 in alum, four times at ≥4 week intervals. Splenocytes and Ag8 myeloma cells, at a ratio of 2:1, were fused with 50% polyethylene glycol-4000 and grown in selection medium (OptiMEM I with 5% fetal calf serum, azaserine-hypoxanthine, and interleukin-6) for 10 d. Colonies from wells that tested positive by ELISA were isolated and expanded in selection medium for 1 week. For monoclonal antibody production, positive clones were maintained in OptiMEM with 5% fetal calf serum and 50–100U ml^–1^ of interleukin-6, and the culture supernatant was harvested when cell viability started to decay.

### SDS-PAGE and Western blot analysis

Samples with recombinant protein were analysed for purity by SDS-PAGE using a 12% acrylamide separation gel and colloidal Coomassie blue staining. For Western blots, unstained gels were blotted onto a polyvinylidene difluoride membrane (Merck, Darmstadt, Germany) for 1h at 70V. The membrane was blocked for 1h with TBST-BSA (1% w/v) at room temperature followed by two washing steps with TBST each for 15min. For detection of the His tag, a SuperSignal West HisProbe kit (Thermo Scientific, Vienna, Austria) was used according to the manufacturer’s manual. Luminescence detection was performed using an LAS 3000 mini imaging system (Fujifilm, Düsseldorf, Germany).

Proteins of *C. australis* extracts were separated by SDS-PAGE as described by Schmölzer *et al*. (2011). For Western blots, an IgG polyclonal antibody raised in rabbit against ARA6 from *A. thaliana* (Ueda *et al.*, 2001) was diluted 1:200. The secondary antibody, an anti-rabbit IgG coupled to horseradish peroxidase (Sigma-Aldrich) was used at a concentration of 1:80 000. The monoclonal IgG antibody raised against CaARA6 was diluted 1:200. The secondary antibody, a mouse IgG coupled with alkaline phosphatase, was used at a dilution of 1:20 000 and detected by luminescence as described above.

### Transformation of *Agrobacterium tumefaciens* and transient transfection of *Nicotiana benthamiana* leaves

An aliquot of competent *A. tumefaciens* (200 µl) strain GV3101 carrying pSoup helper plasmid was taken from –80 °C and thawed on ice, followed by addition of 1 µg of circular plasmid and 30min incubation on ice. Subsequently, cells were frozen in liquid nitrogen for 1min and thawed again in a 37 °C water bath. One ml of YEB medium was added and the suspension was incubated for 4h at 28 °C under vigorous shaking. After this regeneration step, 200 µl were plated on YEB plates containing 50 µg ml^–1^ of kanamycin and 25 µg ml^–1^ of gentamycin. Plates were incubated at 28 °C for 2 d.

*N. benthamiana* plants were grown on standard fertilized soil (type ED73) in a growth chamber with a 16/8h light/dark cycle. The temperature was adjusted to 23 °C during light and to 22 °C during the dark phase; relative humidity was set to 60%. Four-week-old plants were used for *Agrobacterium*-mediated transient protein expression.

Two to three loops of plasmid carrying *A. tumefaciens* were taken from a fresh, not overgrown YEB plate supplemented with appropriate antibiotics (50 µg ml^–1^ of kanamycin, 25 µg ml^–1^ of gentamycin) and grown at 28 °C in 10–20ml of liquid YEB medium under continuous shaking (about 200rpm) to late-exponential phase (about 16h). Cultures were harvested by centrifugation (6000*g*, 5min, room temperature) and the pellet was resuspended in 5ml of infiltration buffer [10mM MES/KOH (pH 5.6), 10mM MgCl_2_, 150 µM acetosyringone], followed by photometric determination of bacterial concentration (OD_600_). Bacterial suspensions were mixed to adjust to the following final concentrations: OD_600_=0.5 for *Agrobacterium* carrying either *ARA6* or *ARA7*; OD_600_=0.25 for the P19 repressor. Final suspensions were incubated for 2h at room temperature, followed by infiltration into 4-week-old *N. benthamiana* leaves using a 1ml syringe. Infiltrated leaf areas were marked with a pen to enable precise recognition of infiltrated parts after expression for 3–5 d in a growth chamber with 16/8h light/dark cycle (temperature and humidity as described above).

### *In vivo* staining and inhibitor treatments

Internodal cells were pulse labelled for 5min with 10 µM of the green fluorescent endocytic tracer FM1-43FX [*N*-(3-triethylammoniumpropyl)-4-(4-(dibutylamino)styryl)pyridinium dibromide; Invitrogen; Carlsbad, CA, USA; 500 µM stock solution in dH_2_O] or the red fluorescent endocytic tracer AM4-65 [*N*-(3-triethylammoniumpropyl)-4-(6-(4-(diethylamino) phenyl) hexatrienyl) pyridinium dibromide; Biotium, Hayward, USA; 1mM stock solution in dH_2_O], which is spectrally identical to FM4-64. Tobacco leaf epidermal cells were stained by infiltration of leaves with 10 µM of the red fluorescent FM4-64 [*N*-(3-triethylammoniumpropyl)-4-(6-(4-(diethylamino)phenyl)hexatrienyl)pyridiniumdibromide; Invitrogen; 10mM stock solution in DMSO] or with 10 µM of the acidotropic dye LysoTracker Red DND-99 (LTRed; Invitrogen; 1mM stock solution in DMSO).

For inhibitor experiments, leaves were infiltrated with 50 µM cytochalasin D (CD; Sigma; 10mM stock solution in DMSO), 50 µM oryzalin (Riedel-de Haën, Seelze, Germany; 10mM stock solution in DMSO), 500 µM brefeldin A (BFA; Sigma-Aldrich; 70mM stock solution) and 200 µM WM (Enzo, Farmingdale, USA; 10mM stock solution in DMSO). These concentrations affected the cytoarchitecture of epidermal cells within 2h of treatment. Lower concentrations had either no effect or required much longer incubation times with possible non-specific effects.

All dyes and inhibitors were diluted with artificial fresh water. Controls containing up to 2% DMSO had no visible effect on cytoarchitecture, cytoplasmic streaming, or organelle dynamics.

### Immunofluorescence

The immunofluorescence procedure was as described by Schmölzer *et al*. (2011). For localization of ARA6-labelled organelles, an IgG antibody raised in rabbit against ARA6 from *A. thaliana* (Ueda *et al.*, 2001) was used at a dilution of 1:200 and combined with an Alexa Fluor 546-conjugated anti-rabbit IgG produced in goat (Invitrogen) and diluted 1:1000. Rabbit pre-immune serum (Sigma-Aldrich) at the same concentration as the primary antibody was used as a negative control. The mouse monoclonal antibody against CaARA6 was diluted 1:10 and combined with a CF488A-conjugated anti-mouse IgG (Sigma-Aldrich) used at a dilution of 1:1000. For comparison of anti-ARA6-labelled organelles with structures stained by endocytic tracers, cells were pulse labelled with 10 µM of the fixable probe FM 1-43FX or AM4-65 and fixed in glutaraldehyde after a 30min wash in artificial fresh water.

### Confocal laser-scanning microscopy (CLSM)

CLSM was carried out using a Leica (Mannheim, Germany) TCS SP5 coupled to a DMI 6000B inverted microscope. Laser settings were as described by Schmölzer *et al*. (2011).

Live internodal cells were mounted in artificial fresh water with or without dye or inhibitor. Leaf sections were either infiltrated with artificial fresh water (with or without dye or inhibitor; see above) or mounted in perfluorodecalin (Littlejohn *et al.*, 2010) in order to fill the intercellular air spaces.

Appropriate controls were made for all single and double labelling experiments in order to exclude bleed through and to distinguish signals from background fluorescence. Co-localizations were studied with sequential scan settings to avoid cross-talk between channels.

Observation times were usually restricted to 2h in order to avoid toxic effects or redistribution of fluorescent dyes. Organelle dynamics were studied by analysing time series taken at minimum laser intensity and pixel time in order to avoid photobleaching and a stress response.

All images presented in this study are single sections unless otherwise stated. Images produced by the LSM software were further processed with Adobe Photoshop (Adobe Systems Inc.).

### Image analysis, size measurements, and statistics

The velocity of organelles was calculated from time series taken at 1.3 s intervals from three different cells and data are given as means±SD. Displacements ≤1.5 µm s^–1^ were considered as oscillating or Brownian motion and were not included in the measurements. ImageJ (http://rsbweb.nih.gov/ij) was used to count and measure ARA6-labelled organelles. Parametric and non-parametric tests were performed with SigmaStat v.3.11 (Systat Software, Erkrath, Germany) in order to reveal significant differences (*P*≤0.05) in size (given as the largest cross-section or diameter). Graphs were generated with SigmaPlot v. 9.01 (Systat Software).

### Electron microscopy

Branchlet internodal cells of *C. braunii* were cryofixed in a Leica EMPACT high-pressure freezer, freeze-substituted in a Leica EM AFS freeze-substitution apparatus, embedded in LR Gold Resin (London Resin Company, Theale, UK) and processed for immunolabelling as described by Schmölzer *et al*. (2011). The primary antibody against AtARA6 was used at a dilution of 1:10 in blocking solution; rabbit pre-immune serum at the same dilution was used as a negative control. A 10nm gold-conjugated anti-rabbit IgG produced in goat (Sigma-Aldrich) and diluted 1:40 in blocking solution was used as a secondary antibody. Micrographs in elastic bright-field mode were taken with a LEO 912 transmission electron microscope (Zeiss) equipped with an in-column energy filter.

## Results

### Identification, molecular characterization, and phylogenetic analysis of an ARA6-like protein in *Chara*

In order to find out whether an ARA6-like protein is present in characean green algae, we sequenced a cDNA library of *C. australis* with a Roche GS FLX system and searched for sequences similar to *A. thaliana ARA6* (*AtARA6*). Comparisons resulted in a highly similar contig of *Chara* composed of 120 aa representing the C-terminal part of the protein. Based on the sequence of *C. braunii*, obtained from an independent sequencing project, ARA6 primers were designed for cloning and sequencing of *CaARA6*. Amplification resulted in a 714bp sequence (accession number HF968767) encoding a protein of 237 aa and a calculated molecular mass of 25.4kDa. With the exception of single amino acids, the sequence of *C. australis* was almost identical to the *C. braunii* sequence (Supplementary Fig. S2A at *JXB* online).

**Fig. 1.**
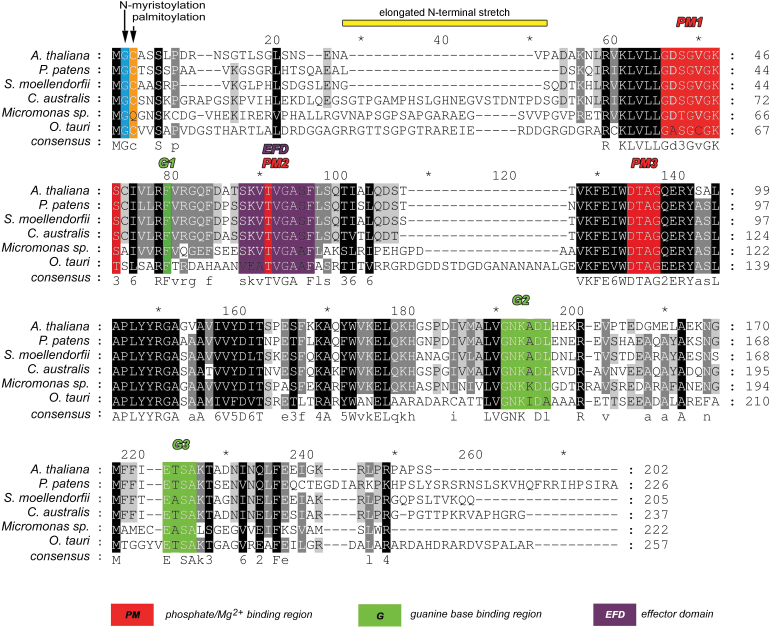
Protein sequence alignment of ARA6 members. Multiple sequence alignment of amino acids from CaARA6 with other organisms was performed using ClustalW. Numbering of amino acid residues begins at the first methionine. Identical residues are highlighted in black and conserved domains are displayed in different colours.

The sequence of *CaARA6* is shown in Fig. 1. Alignment of this sequence with that of *A. thaliana*, *Physcomitrella patens* and *S. moellendorffii* revealed several highly conserved regions, which are partially involved in phosphate and guanine base binding or representing the effector domain. A putative *N*-myristoylation and palmitoylation site for membrane anchoring was present at Gly2 and Cys3. Apart from these conserved domains, the *Chara* sequence showed an extra stretch of about 30 aa in the N-terminal region (aa 29–54). The unique N-terminal extension and absence of C-terminal prenylation sequences are characteristic for this plant-specific family of RAB5-like proteins, as shown in a comparison of ARA6 and ARA7 from *C. australis* and *A. thaliana* (Supplementary Fig. S2B).

**Fig. 2.**
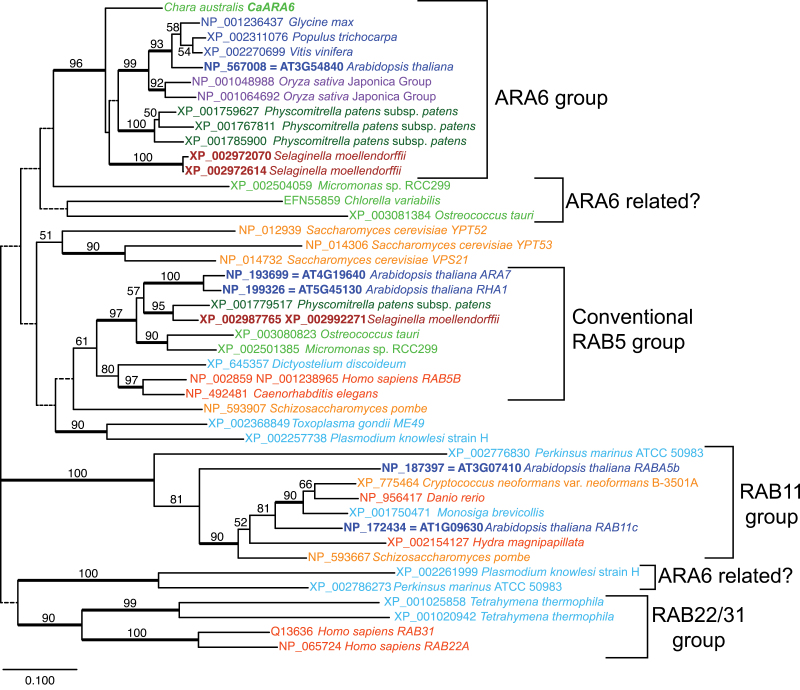
Neighbour-joining tree of 44 ARA6-related genes. Each terminal gene is shown with the accession number and the name of the organisms the gene was isolated from. For *A. thaliana* genes, the AGI code is shown in addition to the accession number. Gene names are written for *C. australis*, *A. thaliana*, human, mouse, and yeasts. The colour represents the taxonomic position of the organism according to the NCBI taxonomy database: blue, eudicots; purple, monocots; brown, other vascular plants (e.g. lycophytes); green, bryophytes; lime green, green algae; orange/red, animals; dark orange, fungi; light blue, other eukaryotes. Bootstrap probabilities >50% are shown on each branch. Branches with low bootstrap value <50% are drawn with dotted lines and branches with high support ≥90% are drawn with thick lines.

A database search of sequenced algae revealed additional potentially ARA6-related sequences in the prasinophyte green algae *Micromonas* sp. RCC299 (XP_002504059) and *Ostreococcus tauri* (XP_003081384), and in alveolata species *Plasmodium knowlesi* strain H (XP_002261999) and *Perkinsus marinus* ATCC 50983 (XP_002786273). In phylogenetic analyses, *Chara* ARA6 and land plant ARA6s formed a clade (Fig. 2). The *Micromonas* gene was placed sister to that clade, and the *Ostreococcus tauri* gene XP_003081384 was placed with a *Chlorella* gene EFN55859 at a further basal position with low bootstrap support. The phylogenetic relationship of alveolata sequences in regard to ARA6 was unclear, presumably due to the large divergence time and short sequences. Except for the *Chlorella* gene EFN55859, which is partial and lacks the start information, the sequences including those from alveolata species shared N-terminal extension starting with MGCXXS (M, G, and S are completely conserved, C is substituted with Q only in *Micromonas* XP_002504059).

### Biochemical characterization of CaARA6

**Fig. 3.**
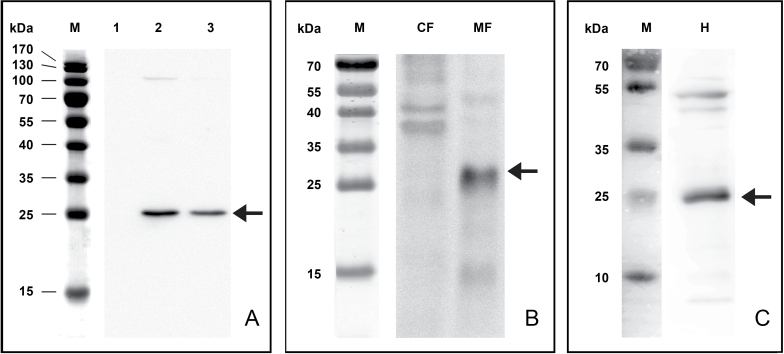
Western blots of recombinant CaARA6 (A) and of *C. australis* protein extracts (B, C). (A) His-tagged CaARA6 purified with a Ni-NT affinity column and detected with His-specific antibody. Lane M, molecular mass marker; lane 1, lysate of *E. coli* before IPTG induction; lane 2, lysate of *E. coli* 20h after IPTG induction; lane 3, CaARA6-His purified on chelating column. (B, C) Antibodies against AtARA6 (B) and CaARA6 (C), with arrows labelling a prominent band with a molecular mass of about 25 kD (arrows). CF, cytosolic fraction; MF, membrane fraction; H, homogenate.

In order to get information on the biochemical properties of CaARA6, the open reading frame of *CaARA6* was cloned into an *E. coli* expression vector for production of the His-tagged recombinant protein. The recombinant protein was detected with a His-specific antibody, which recognized a 27kDa protein in Western blots (Fig. 3A). The high sequence similarity of the *Chara* protein with that of *A. thaliana* allowed the use of a polyclonal antibody raised against AtARA6 (Ueda *et al.*, 2001). In addition, we developed a monoclonal antibody against CaARA6. In Western blots of *Chara* extracts, both antibodies detected a single band with an apparent molecular mass of about 25kDa (Fig. 3B, C).

**Fig. 4.**
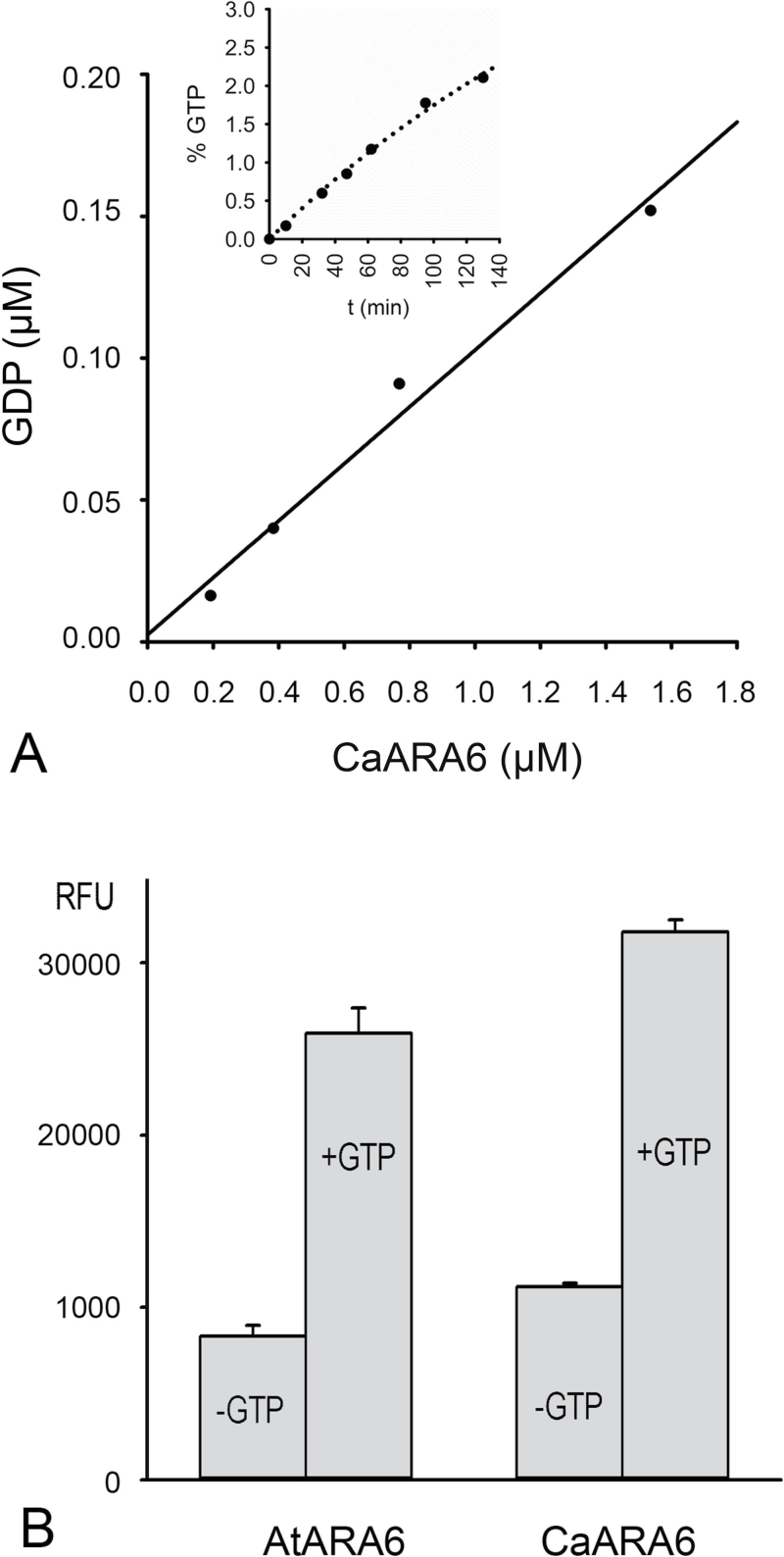
GTP hydrolyzing activity of recombinant ARA6. (A) GTPase activity of CaARA6 CaARA6 plotted as GDP production in relation to enzyme concentration (0.19–1.53 µM). Purified recombinant protein was incubated for 1h in the presence of 10 µM GTP (25 µl reaction mixture volume), and the reaction was terminated by the addition of an equal volume of ‘stop and detect reagent’ and the free fluorescent GDP–Alexa Fluor 594 was measured as indicated in Materials and Methods. The inset shows the time course of GDP production in the presence of 10 µM GTP and 0.8 µM recombinant CaARA6. (B) GTPase activity measured at 120min of incubation as relative fluorescence of GDP–Alexa Fluor 594 for recombinant AtARA6 and CaARA6 (1.6 µM each) in the presence and absence of 10 µM GTP. Data are given as means±SEM.

The GTPase activity of CaARA6 was measured with a highly sensitive fluorescence assay and compared with that of AtARA6. Figure 4A shows GDP production after 1h incubation and a typical time course of GDP production at a given enzyme concentration (Fig. 4A, inset). Figure 4B shows the GTPase activity of CaARA6 in comparison with that of AtARA6. The low amounts of GDP measured when GTP was omitted from the reaction mixture probably originated from GDP, which was bound to the enzymes and was passively (non-enzymatically) released during incubation. This indicated that both CaARA6 and AtARA6 have intrinsic GTPase activity (~0.005 and 0.004mol min^–1^ mol protein^–1^, respectively), which can be further enhanced by activators (cf. Ueda *et al.*, 2001).

### Transient expression in tobacco leaf epidermal cells and co-localization with AtARA6

As characean green algae transfection methods were unavailable, we transiently expressed fluorescently tagged *CaARA6* in epidermal cells of tobacco leaves (*N. benthamiana*) under the control of the *A. thaliana* ubiquitin-10 promoter (Norris *et al.*, 1993). Either GFP or mCherry was fused to the C terminus of both *C. australis* and *A. thaliana* ARA6 in order to determine the localization of these proteins.

**Fig. 5.**
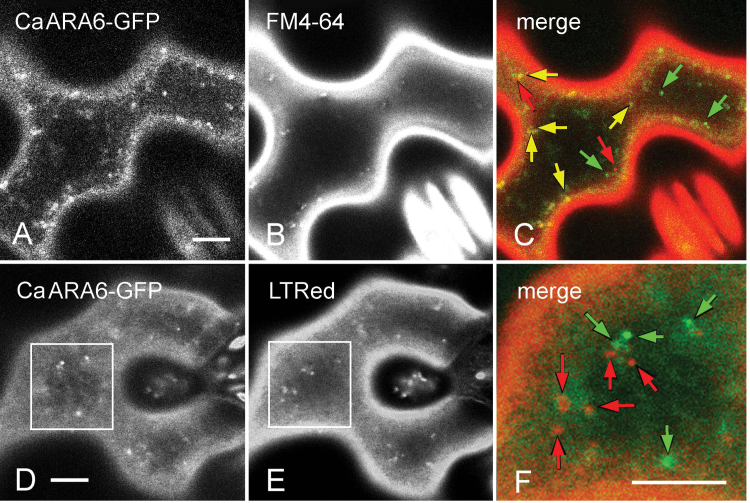
Transient expression of CaARA6–GFP in tobacco leaf epidermal cells and comparison with fluorescent dyes. (A–C) Comparison of CaARA6–GFP with FM4-64, an endocytic tracer. The fluorescence of CaARA6–GFP-positive organelles (A) partly overlapped with that of FM4-64-stained particles (B) 20min after pulse labelling. In the merged image (C), green arrows indicate organelles carrying the CaARA6–GFP fluorescence only, red arrows indicate organelles labelled by FM4-64 only, and yellow arrows indicate co-localizations. (D–F) Comparison of CaARA6–GFP with LTRed, an acidophilic dye. CaARA6–GFP-labelled organelles (D) were not stained by LTRed (E). The merged image (F) is a higher magnification of the inset shown in (D) and (E). Organelles fluoresced either green (green arrows) or red (red arrows). Bars, 10 µm.

**Fig. 6.**
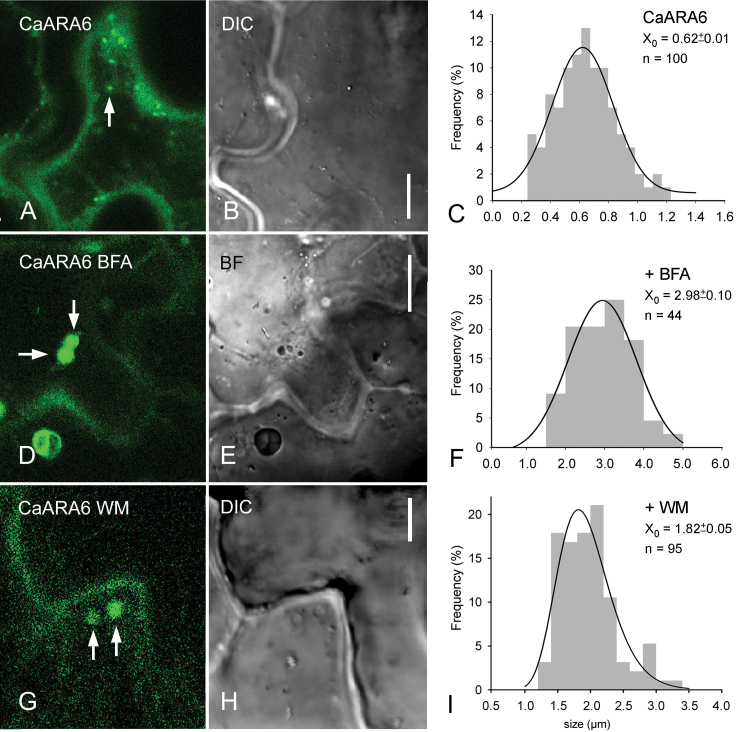
Effect of BFA and WM on CaARA6–GFP transiently expressed in tobacco leaf epidermal cells. (A–C) Untreated control leaf epidermal cell with small punctuate organelles (arrow) (A), differential interference contrast (DIC) image (B) and size–frequency distribution plot (C). (D–F) Leaf infiltrated with 500 µM BFA. Arrows indicate BFA compartments in the fluorescence (D) and in the bright-field (BF) image (E). (F) is the size–frequency distribution of fluorescent organelles. (G–I) Leaf infiltrated with 200 µM WM. Arrows indicate WM compartments in the fluorescence image (G) and in the DIC image (H). (I) is the size–frequency distribution of fluorescent organelles. Bars, 10 µm (A, B, D, E); 5 µm (G, H). (This figure is available in colour at *JXB* online.)

*In vivo* analysis of the localization of the CaARA6–GFP fusion protein by CLSM revealed its location on small, punctuate organelles with diameters ranging between 0.2 and 1.2 µm (mean±SD: 0.62±0.01; *n*=100; Fig. 5A, D; Fig. 6A–C). These organelles moved along linear or curved tracks with a mean velocity of 3.4±1.1 µm s^–1^ (*n*=45), interrupted by oscillating motions (defined as displacements ≤1.5 µm s^–1^) (Supplementary Fig. S3A, Supplementary Video S1 at *JXB* online). Oryzalin, a microtubule-depolymerizing drug, had no significant effect on trajectories and velocity (3.4±1.2 µm s^–1^; *n*=38; Supplementary Fig. S3B), whereas CD completely arrested directed movements indicating actin-dependent dynamics of CaARA6–GFP-labelled organelles (Supplementary Fig. S3C, Supplementary Video S2 at *JXB* online). The CaARA6–GFP fluorescence partially overlapped with that of FM4-64-stained endosomes (Fig. 5A–C). CaARA6–GFP was not detectable in organelles stained with the acidotropic LTRed (Fig. 5D–F). Treatment with BFA induced the formation of larger compartments with a mean size of 2.98±0.10 µm (*n*=44) showing CaARA6–GFP fluorescence (Fig. 6D–F) and WM-treated cells formed characteristic vacuole-like compartments with a mean diameter of 1.82±0.05 µm (*n*=95; Fig. 6G–I). The decreased abundance or even absence of small fluorescent particles suggested that BFA and WM compartments formed at the expense of smaller organelles.

**Fig. 7.**
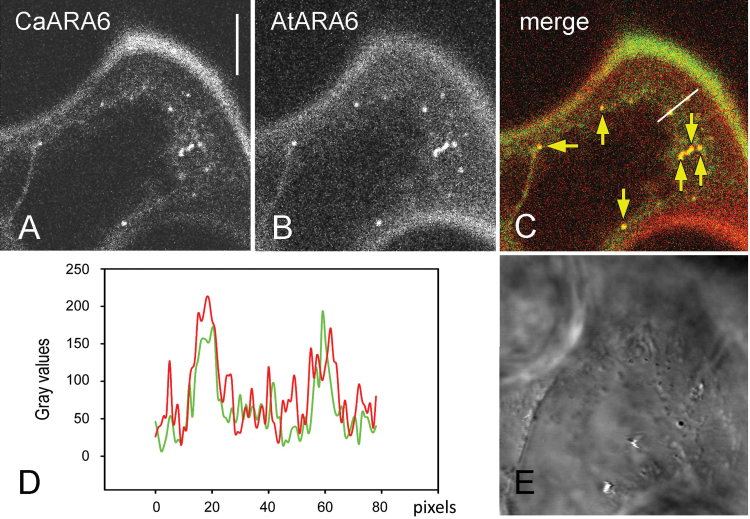
Co-expression of CaARA6–GFP with AtARA6–mCherry in epidermal cells of tobacco leaves. (A, B) CaARA6–GFP-labelled organelles (A) co-localized with AtARA6-labelled particles (B). (C) Merged image. (D) Graph of the grey values plotted along the white line in (C). (E) DIC image. Bar, 10 µm.

In order to get more information about the identity of the CaARA6–GFP-localizing organelles, we compared the distribution of CaARA6 with that of AtARA6 using pGI-*AtUBQ10p::AtARA6::GFP* with pGI-*AtUBQ10p::CaARA6::mCherry* and in separate experiments pGI-*AtUBQ10p::AtARA6::mCherry* with pGI-*AtUBQ10p::CaARA6::GFP*. When expressed alone in tobacco leaf epidermal cells, tagged AtARA6 localized to organelles that had a similar dynamic behaviour and a similar size (Supplementary Fig. S4 at *JXB* online) as the fluorescent CaARA6 particles. In epidermal cells of untreated leaves, co-expression of both proteins resulted in an overlap of fluorescent compartments varying between 50 and 100% (Fig. 7A–E; mean 75.1%, *n*=507 organelles from 39 images) and probably reflecting the degree of expression. Co-localization of BFA and WM compartments was 100% (*n*=13 from eight images; Supplementary Fig. S5A–H at *JXB* online). These data suggested that tagged ARA6 from *C. australis* and *A. thaliana* localized to the same organelles in tobacco epidermal cells. These organelles have previously been identified as MVEs (Haas *et al*., 2007), consistent with the observation that CaARA6-positive compartments do enlarge upon WM treatment.

### Point mutations consistent with membrane anchoring via palmitoylation and myristoylation and GTPase activity of CaARA6

The high sequence similarity and co-localization with AtARA6 indicated that CaARA6 has similar properties to RAB5 GTPase in flowering plants. In order to characterize further the membrane-binding properties of CaARA6, we introduced point mutations that paralleled those used by Ueda *et al.* (2001) in AtARA6 and transiently expressed the GFP-tagged mutants in tobacco leaf epidermal cells.

As CaARA6, like its homologue AtARA6, has putative *N*-myristoylation and palmitoylation sites for membrane anchoring at Gly2 and Cys3, we investigated whether point mutations at these sites altered the membrane binding and consequently the subcellular localization of CaARA6. The GFP fusion of CaAra6^C3S^ (a mutant having an amino acid substitution in Cys3, which impairs palmitoylation) was localized in the cytosol and at slowly moving organelles clearly distinct from CaARA6-positive endosomes because of their larger diameter (1.13±0.19 µm; *n*=33) and their co-staining with LTRed (Supplementary Fig. S6A–I at *JXB* online). The CaAra6^G2A_C3S^ mutant, which lacks both putative *N*-myristoylation and palmitoylation sites, was detected in the cytosol and nucleus only (Supplementary Fig. S6J–L), showing a distribution pattern similar to the mutant AtAra6^G2A_C3S^. These results suggested that N-terminal fatty acylation is of stringent necessity for proper membrane anchoring of CaARA6.

In addition, we investigated whether, as for AtARA6, nucleotide binding plays a role in the subcellular localization of CaARA6 and introduced point mutations that affect the binding affinity for either GTP or GDP or both. The nucleotide-free mutant CaAra6^N172I^ (corresponding to N147I in AtARA6) was found in the plasma membrane and the cytosol (Supplementary Fig. S7A–C at *JXB* online), suggesting that nucleotide binding is necessary for localization at endosomes. The GDP-bound CaAra6^S73N^ (corresponding to S47N in AtARA6) was present in the cytosol and, additionally, at large, slowly moving organelles (1.11±0.22 µm; *n*=81) stained by LTRed (Supplementary Fig. S7D–I) and probably identical to those observed with CaAra6^C3S^. The constitutively active, GTP-bound mutant CaAra6^Q118L^ appeared at the plasma membrane, in the cytosol, on ring-like structures resembling WM vesicles and LTRed-positive punctate organelles (Supplementary Fig. S8 at *JXB* online). The diameter of these organelles was 1.03±0.19 µm (*n*=54), i.e. significantly larger than that of GFP-labelled CaARA6 particles (Fig. 6C) and also significantly smaller than those of the CaAra6^C3S^ and CaAra6^S73N^ mutant fusions (*t*-test; *P*<0.05). Notably, the tonoplast, which has been described to be a target of Ara6^Q93L^ in *A. thaliana* (Ueda *et al.*, 2001; Ebine *et al*., 2011), was never stained by the CaAra6^Q118L^ mutant fusion.

### CaARA6 localizes to multivesicular endosomes, the TGN, and the plasma membrane in *Chara* internodal cells

**Fig. 8.**
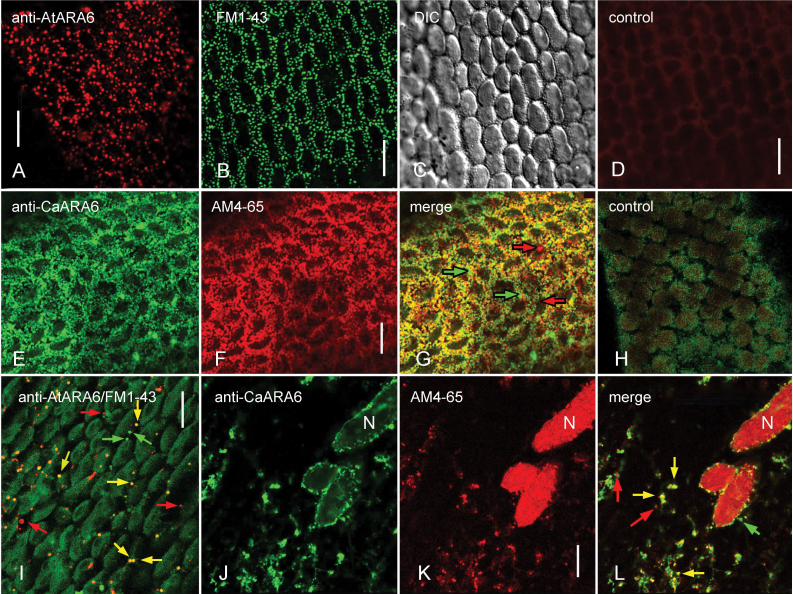
ARA6 immunofluorescence in internodal cells of *C. australis* in comparison with the staining pattern of endocytic tracers. (A–C) Punctuate pattern in the cortex obtained by immunofluorescence with antibody against AtARA6 (A) and by *in vivo* FM1-43-staining (B; C is the corresponding DIC image). (D) Immunofluorescence without primary antibody (negative control for A). (E–G) ARA6 epitope visualized by immunofluorescence with an antibody against CaARA6 (E) present at charasomes stained with AM4-65 before immunolabelling (F; G is the merged image). (H) Immunofluorescence without primary antibody (negative control for E) and autofluorescence of chloroplasts. (I) Endoplasmic organelles visualized by immunofluorescence with anti-AtARA6 (red fluorescent) and by staining with FM1-43 (green fluorescent). (J–L) Endoplasmic organelles visualized by immunofluorescence with anti-CaARA6 (J) and with AM4-65 (K; L is the merged image). N, nucleus. Yellow arrows indicate co-localizations, red and green arrows indicate non-overlapping structures. Bars, 10 µm.

We next determined the localization of CaARA6 in *Chara* internodal cells. Immunofluorescence of internodal cells with a polyclonal antibody against AtARA6 and a monoclonal antibody against CaARA6 revealed punctuate structures in the cortex (Fig. 8A, E) and in the endoplasm (Fig 8I, J). The cortical structures had a similar size and distribution as the convoluted plasma membrane invaginations (charasomes), which can be stained in living cells using fluorescent plasma membrane dyes and endocytic tracers (Fig. 8B, C; Schmölzer *et al.*, 2011). In order to find out whether the ARA6 epitope co-localized with endocytic tracers, we treated cells with the fixable FM1-43 or AM4-65 before immunolabelling. In the cortex, the distribution of the ARA6-positive structures overlapped with that of the AM4-65-stained charasomes and only very few organelles were not co-localized (Fig. 8E–G). In the endoplasm, many of the ARA6-positive structures were also stained by FM1-43 or AM4-65, but organelles carrying only one type of fluorescence were also present (Fig. 8I–L). However, the fluorescence of FM1-43- or AM4-65-stained organelles after the immunolabelling procedure varied considerably according to the degree of permeabilization and, consequently, the degree of co-localization with ARA6 also showed variation. Specific labelling by the polyclonal anti-AtARA6 antibody was confirmed in negative controls using pre-immune serum instead of the first antibody (compare Supplementary Fig. S9C and D at *JXB* online). Incubation with secondary antibody alone gave no signal (Fig. 8D, H).

The antibody raised against AtARA6 was also suitable for immunolabelling of EM sections. Internodal cells of *C. australis* are too large for high-pressure freezing and chemical fixation does not adequately preserve their cytoplasm. We therefore used the smaller branchlet internodal cells of *C. braunii* after confirming that immunofluorescence with anti-AtARA6 antibody labelled the same structures as in *C. australis* (Supplementary Fig. S9A, B).

**Fig. 9.**
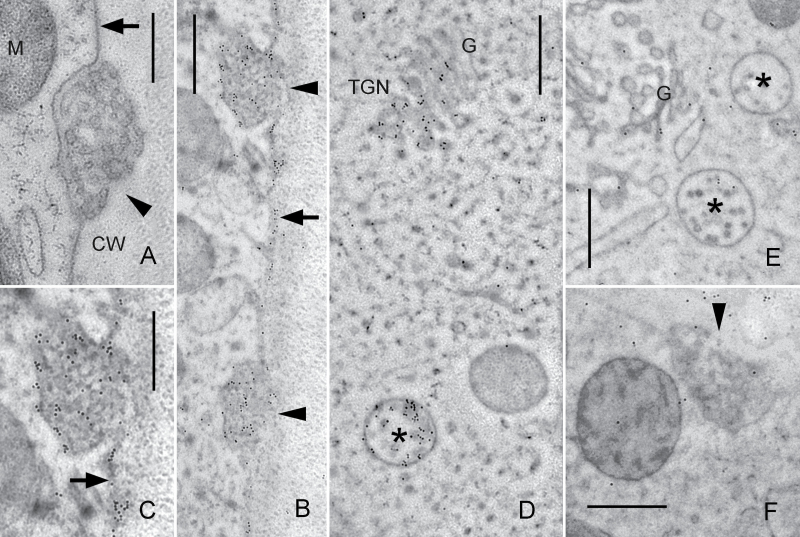
Immunodetection of the ARA6-related protein on EM sections of internodal cells of *C. braunii*. (A) Unstained longitudinal section. Smooth plasma membrane (arrow) and a charasome (convoluted plasma membrane; arrow head) are seen adjacent to the cell wall (CW). M, mitochondrion. (B–D) Immunogold labelling of ARA6. Accumulation of gold particles is seen in charasomes (arrow heads) and at smooth plasma membrane regions (arrows; C is an enlarged detail of B), in multivesicular endosomes (asterisks) and at the TGN. G, Golgi body. (E, F) Negative controls with pre-immune serum instead of primary antibody. A few immunogold particles are randomly distributed over the cytoplasm and the cell wall. Bars, 500nm.

Figure 9A shows an electron microscopical section from the periphery of a high-pressure frozen and cryosubstituted internodal cell. Smooth plasma membrane regions alternate with convoluted domains, the charasomes, which are often located in close proximity to cortical chloroplasts and mitochondria. Immunolabelling with anti-AtARA6 revealed accumulation of gold beads along smooth plasma-membrane regions, at the inner cytoplasmic surface of charasomes, and within charasomes (Fig. 9B, C). In the endoplasm, gold beads were abundant at MVEs and at the TGN (Fig. 9D). These organelles probably corresponded to the endoplasmic particles labelled by immunofluorescence. Far fewer and randomly distributed gold beads were present on sections incubated with pre-immune serum instead of the primary antibody (negative controls; Fig. 9E, F).

## Discussion

### CaARA6 highly similar to AtARA6

ARA6 is a unique type of RAB5 GTPase that has hitherto been considered to be specific for land plants (Ebine *et al.*, 2012). Here, we have provided evidence that an ARA6-like protein with a similar sequence to AtARA6 is present in the characean green algae and we confirmed that this protein had intrinsic GTPase activity. Sequence alignments of the *Chara* protein with that of land plants showed highly conserved regions in the substrate-binding or effector domains and the typical putative *N*-myristoylation and palmitoylation site at the N-terminal glycine and cysteine. An additional 27 aa region at the N terminus, however, was absent in ARA6 members from land plants.

Outside streptophytes, the phylogenetic relationships of ARA6-related proteins are unclear. Two prasinophyte species, *Micromonas* sp. RCC299 and *O. tauri*, have RAB5-related proteins other than the RAB5 orthologues. These proteins share the characteristic N-terminal sequence MGCXXS that was not used in the phylogenetic analysis. The MGCXXS N-terminal sequence is also found in alveolata species *Plasmodium knowlesi* strain H and *Perkinsus marinus* ATCC 50983. The divergence of ARA6 and RAB5 probably occurred in a very early phase of eukaryotic evolution. Notably, in this group of RAB proteins, animals and fungi also have two large clusters. Animal have RAB5 and RAB22/RAB31 clusters and fungi have VPS21 and YPT52 (*Saccharomyces cerevisiae* gene name). The ARA6 homologues in *Chlamydomonas reinhardtii*, *Micromonas pusilla* CCMP1545, and *Ostreococcus lucimarinus* CCE9901 were not found, whereas RAB5 homologues were found. This implies that ARA6 homologues tend to be lost more easily in organisms with small genome sizes, including prasinophytes.

### Similar localization of CaARA6 and AtARA6 when transiently expressed in tobacco

When simultaneously expressed in tobacco leaf epidermal cells, CaARA6 co-localized with organelles carrying AtARA6. AtARA6-positive organelles were identified as MVEs by immunolabelling of EM sections and by comparison of tagged AtARA6 with other MVE markers in *A. thaliana* (Haas *et al*., 2007). These organelles carrying CaARA6 and AtARA6 fused to larger compartments upon treatment of cells with WM, which is consistent with the formation of WM compartments by homotypic fusion of MVEs (Wang *et al.*, 2009). Tobacco leaf epidermal cells also formed BFA compartments carrying both the CaARA6 and the AtARA6 fluorescence, as described in root epidermal cells of *A. thaliana* (Ebine *et al*., 2011).

Our data about the subcellular localization of GFP-tagged CaARA6 mutants were similar to those obtained with point mutations in AtARA6 (Ueda *et al.*, 2001; Goh *et al.*, 2007). The presence of the GFP-tagged mutant CaAra6^G2A/C3S^ exclusively in the cytosol and the nucleus but not in membrane-bound structures strengthens the hypothesis that the putative *N*-myristoylation and palmitoylation sites present at Gly2 and Cys3 in CaARA6 are essential for proper membrane anchoring, as was found for AtARA6 (Ueda *et al.*, 2001).

The nucleotide-free mutant GFP-tagged CaAra6^N172I^ stained only the plasma membrane and was absent from mobile endosomal organelles, probably because it cannot cycle between the GDP- and GTP-bound forms (Ueda *et al.*, 2001). The constitutively active, GTP-locked mutant CaAra6^Q118L^ resided predominantly at the plasma membrane, at ring-like structures, and occasionally at slowly moving organelles about 1 µm in size (cf. Ueda *et al.*, 2001; Ebine *et al*., 2011). This finding is consistent with a functional localization of CaARA6 to the plasma membrane, whereas the presence of the ring-like structures could be attributed to an increased homotypic vesicle fusion activity of the CaAra6^Q118L^ mutant (Stenmark *et al.*, 1994; Roberts *et al*., 1999). A notable difference of CaAra6^Q118L^ as compared with the corresponding point mutation in AtARA6 was the absence of the fluorescent signal at the tonoplast. The different behaviour may be due to the extra stretch of amino acids at the N-terminal region of CaARA6.

Interestingly, the GFP-tagged CaAra6^C3S^, CaAra6^S73N^, and CaAra6^Q118L^ mutants co-localized with LTRed, whereas organelles carrying the non-mutated GFP-tagged CaAra6 were never stained with this dye. LTRed selectively accumulates in acidic compartments including lysosomes, autolysosomes, and vacuoles (Derrien *et al.*, 2012). Our data may therefore indicate that some of the mutated GFP-tagged CaAra6 proteins become degraded in autophagosomes.

None the less, the high sequence similarity, the co-localization of tagged CaARA6 with tagged AtARA6, and the comparable distribution of GFP-tagged mutants suggest similar functions for both proteins when expressed transiently in tobacco epidermal cells: endosomal trafficking to the plasma membrane and a trafficking event around MVEs.

### Different localization and function of ARA6-like protein in *Chara* internodal cells

The localization of CaARA6 in *Chara* cells was studied with antibodies raised against AtARA6 (Ueda *et al.*, 2001) and against CaARA6. Both immunofluorescence and immunogold labelling revealed binding of the antibody against AtARA6 on MVEs as expected from the co-localization in *N. benthamiana* and immunogold studies in *A. thaliana* (Haas *et al*., 2007). In *Chara* internodal cells, however, immunolabelling was not restricted to MVEs. The antibodies recognized additional epitopes at the plasma membrane, including the charasomes, and at the TGN (highlighted in red in Supplementary Fig. S1B). Hence, the ARA6 epitope in internodal cells is present in the plasma membrane and at organelles considered to represent early and late endosomes (e.g. Viotti *et al.*, 2010). In the green alga *Chara*, this ARA6-like GTPase could therefore have a broad function in endosomal trafficking including ‘classical’ endocytosis of the plasma membrane via the TGN and MVEs. It is also possible that CaARA6 is involved in membrane fusion at the plasma membrane (and the TGN and MVEs) in conventional and/or endosomal secretion, considering its substantial localization on the plasma membrane. On the other hand, AtARA6 has a more restricted localization at the MVE and a more specialized function in alternative endosomal trafficking routes and in salt stress response in *A. thaliana* (Ebine *et al.*, 2011). Thus, it would be an interesting possibility that functions of ARA6 members are diversified among plant lineages, while further studies of ARA6 members in other plant lineages such as the bryophyte and lycophyte are apparently needed.

In *A. thaliana*, the constitutively active AtARA6 mutant could be visualized at the plasma membrane of root epidermal cells and was shown to form discrete speckles following NaCl treatment (Ebine *et al.*, 2011). In characean internodal cells, wild-type ARA6-like proteins are abundant at the plasma membrane, even under normal, unstressed conditions. We did, however, never observe CaARA6–GFP at the plasma membrane of transformed tobacco leaf epidermal cells. Most probably, CaARA6 can only associate with *Chara* plasma membrane (or TGN) at the steady state under the control of the *Chara* cytoplasm. We cannot exclude the possibility, however, that the fluorescent tag alters the association with membranes in *N. benthamiana.* Altered transport of soluble vacuolar proteins upon expression of fluorescently tagged mutant AtARA6 and AtARA7 has been described in *Nicotiana* leaf epidermal cells (Kotzer *et al.*, 2004; Bottanelli *et al.*, 2011). AtARA6-positive organelles in root epidermal cells of *A. thaliana* do not carry known TGN markers (Ebine *et al*., 2011). Recent data also indicate that MVEs mature from the TGN in *A. thaliana* and that the V-ATPase inhibitor concanamycin A causes fusion of MVEs with the TGN and redistribution of AtARA6 and AtARA7 to the TGN (Scheuring *et al.*, 2011). Immunolabelling in *Chara* suggests that CaARA6 locates to both the TGN and MVEs, which, on the one hand, strengthens the hypothesis of MVE maturation from the TGN, and on the other hand, makes it unlikely that our observations are labelling artefacts.

### Is CaARA6 required for charasome formation and plasma membrane repair?

Our immunofluorescence and EM data indicated that CaARA6 localizes to the plasma membrane and to charasomes in internodal cells of *C. australis* and *C. braunii*. Charasomes are structured plasma membrane elaborations involved in the acidification of the environment, which increases the efficiency of 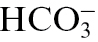 utilization and photosynthesis (see Schmölzer *et al.*, 2011, for references). A similar increase in plasma membrane area is found in ‘transfer cells’, which are widespread over the whole plant kingdom including mosses, ferns, and seed plants (Gunning and Pate, 1969; Offler *et al.*, 2003). Charasomes have been described to develop from tubular invaginations (Lucas and Franceschi, 1981) or extensions of the plasma membrane (Chau *et al.*, 1994). Irrespective of how charasomes develop, it is clear that exocytosis and endocytosis at these domains have to be differentially regulated in comparison with smooth plasma membrane regions. In this respect, it is interesting to note that vps9a-1 mutants form plasma membrane-associated vesicular structures that strikingly resemble charasomes (Goh *et al.*, 2007). VPS9a is a guanine nucleotide exchange factor that activates all members of the RAB5 family in *A. thaliana*. The immunogold data suggest that CaARA6 is present not only at the inner surface of the charasomes but also within the charasomes where vesicular endocytosis is not possible because of steric reasons. It is thus possible that CaARA6 is involved in establishing and maintaining the complex tubular architecture of charasomes.

FM dyes are widely used as endocytic tracers. Unlike in higher plant cells, where the FM dyes disappear from the plasma membrane after pulse labelling, the plasma membrane of internodal cells retains the FM fluorescence for several hours. It has been suggested that this is due to recycling via putative FM-stained early endosomes (Klima and Foissner, 2008). Recently, we found that FM-stained vesicles are involved in the early stages of wound healing (Klima and Foissner, 2011). In view of the data presented here about the presence of ARA6-like protein at the plasma membrane and at the TGN, which is at the intersection between endocytic and secretory pathways in flowering plants (Foresti and Denecke, 2008), it is possible that CaARA6 located at TGN-derived vesicles is involved in plasma membrane repair. This is supported by recent findings implicating ARA6 in the mediation of direct transport from endosomes to the plasma membrane, especially under stressed conditions (Ebine *et al*., 2011). Involvement in membrane trafficking under salinity stress has also been described for the ARA6 homologue of *Mesembryanthemum crystallinum* (Bolte *et al*., 2000, 2004).

## Supplementary data

Supplementary data is available at *JXB* online.

Supplementary Table S1. Primer list.

Supplementary Fig. S1. Characean thallus and schematic longitudinal section through internodal cell.

Supplementary Fig. S2. Sequence alignments of different ARA6 and ARA7 proteins.

Supplementary Fig. S3. Actin-dependent dynamics of CaARA6–GFP transiently expressed in leaf epidermal cells of *N. benthamiana*.

Supplementary Fig. S4. Size distribution plot of organelles labelled by AtARA6–GFP in transiently transformed leaf epidermal cells of *N. benthamiana.*

Supplementary Fig. S5. Tagged CaARA6 and AtARA6 co-localize at BFA and WM-induced compartments when expressed in leaf epidermal cells of *N. benthamiana*.

Supplementary Fig. S6. Leaf epidermal cells of *N. benthamiana* expressing GFP-tagged CaAra6^C3S^ and CaAra6^G2A_C3S^.

Supplementary Fig. S7. Leaf epidermal cells of *N. benthamiana* expressing GFP-tagged CaAra6^N172I^ and CaAra6^S73N^.

Supplementary Fig. S8. Leaf epidermal cells of *N. benthamiana* expressing GFP-tagged CaAra6^Q118L^.

Supplementary Fig. S9. Immunofluorescence with a polyclonal antibody against AtARA6 and autofluorescence of chloroplasts in internodal cells of *C. braunii*.

Supplementary Video S1. CaARA6–GFP-labelled organelles in untreated tobacco leaf epidermal cells.

Supplementary Video S2. CaARA6–GFP-labelled organelles in tobacco leaf epidermal cells infiltrated with 50 µM cytochalasin D for 1h.

Supplementary Data

## Abbreviations:

BFA: brefeldin A
CD: cytochalasin D
CLSM: confocal laser-scanning microscopy
DIC: differential interference contrast
EM: electron microscopy
LTRed: LysoTracker Red
MVE: multivesicular endosome
TGN: *trans*-Golgi network
WM: wortmannin.
