# Supplementary material for: Molecular and biochemical analysis of the first ARA6 homologue, a RAB5 GTPase, from green algae
Source: J Exp Bot. 2013 Oct 14;64(18):5553–68. doi: 10.1093/jxb/ert322 (PMC3871812; doi:10.1093/jxb/ert322)
Supplement: Supplementary Data [file supp_64_18_5553__index.html]

Molecular and biochemical analysis of the first ARA6 homologue, a RAB5 GTPase, from green algae — Molecular and biochemical analysis of the first ARA6 homologue, a RAB5 GTPase, from green algae — Supplementary Data 

# Molecular and biochemical analysis of the first ARA6 homologue, a RAB5 GTPase, from green algae

## Supplementary Data

Data files

**Files in this Data Supplement:**

- Supplementary Data - Supplementary Data
- Supplementary Data - Supplementary Data
- Supplementary Data - Supplementary Data
