# Supplementary material for: Molecular and biochemical analysis of the first ARA6 homologue, a RAB5 GTPase, from green algae
Source: J Exp Bot. 2013 Oct 14;64(18):5553–68. doi: 10.1093/jxb/ert322 (PMC3871812; doi:10.1093/jxb/ert322)
Supplement: Supplementary Data [file supp_ert322_jexbot101188_file003.pdf]

Molecular and biochemical analysis of the first ARA6 homolog, a RAB5 GTPase, from green algae

Marion C. Hoepflinger, Anja Geretschlaeger, Aniela Sommer, Margit Hoeftberger, Tomoaki Nishiyama, Hidetoshi Sakayama, Peter Hammerl, Raimund Tenhaken, Takashi Ueda, Ilse Foissner

Supplementary table and figures

Table S 1. Primer list

UBQ10_fwd (KpnI):	5'-GGTACCCGACGAGTCAGTAATAAACG-3'
UBQ10_rev (XhoI):	5'-CTCGAGTGTTAATCAGAAAAACTCAG-3'
GFP6_SmaI_fwd:	5'-CCCGGGATGAGTAAAGGAGAAGAACT-3'
GFP_NotI_rev:	5'-GCGGCCGC**TCA**TGGCGCGCCTTTGTATA-3'
mCherry_SmaI_fwd :	5'-CCCGGGATGGTGAGCAAGGGCGAGGA-3'
mCherry_NotI_rev:	5'-GCGGCCGC**TTA**CTTGTACAGCTCGTCC-3'
AtARA6_HindIII_fwd:	5'-AAGCTTATGGGATGTGCTTCTTCTCT-3'
AtARA6_SmaI_rev:	5'-CCCGGGTGACGAAGGAGCAGGACGAG-3'
CaARA6_HindIII_fwd:	5'-AAGCTTATGGGGTGTAGTAACAGCAA-3'
CaARA6_SmaI_rev:	5'-CCCGGGACCCCTCCCATGAGGGGCTA-3'
ARA6-Fwd-1 (BamHI):	5'-CCGGATCCATGGGGTGTAGTAACAGC-3'
ARA6-Rev-1 (HindIII):	5'-TTAAGCTTTCGACCCCTCCCATGAGG-3'
CaAra6_C3S_fwd:	5'-CGGGGGATCCATGGGGTCCAGTAACAGCAAGCCTGG-3'
CaAra6_C3S_rev:	5'-CCAGGCTTGCTGTTACTGGACCCCATGGATCCCCCG-3'
CaAra6_G2A_C3S_fwd:	5'-CGGGGGATCCATGGCGTCCAGTAACAGCAAGCCTGG-3'
CaAra6_G2A_C3S_rev:	5'-CCAGGCTTGCTGTTACTGGACGCCATGGATCCCCCG-3'
CaAra6_S73N_fwd:	5'-GGGGTAGGAAAGAACTGTATTGTGTTGCGG-3'
CaAra6_S73N_rev:	5'-CCGCAACACAATACAGTTCTTTCCTACCCC-3'
CaAra6_Q118L_fwd:	5'-GGGACACTGCAGGCTTGGAGAGATATGCTTCTCTTGC-3'
CaAra6_Q118L_rev:	5'-GCAAGAGAAGCATATCTCTCCAAGCCTGCAGTGTCCC-3'
CaAra6_N172I_fwd:	5'-GATTGTCATGGCACTTGTTGGCATCAAAGCAGATTTACGAG-3'
CaAra6_N172I_rev:	5'-CTCGTAAATCTGCTTTGATGCCAACAAGTGCCATGACAATC-3'

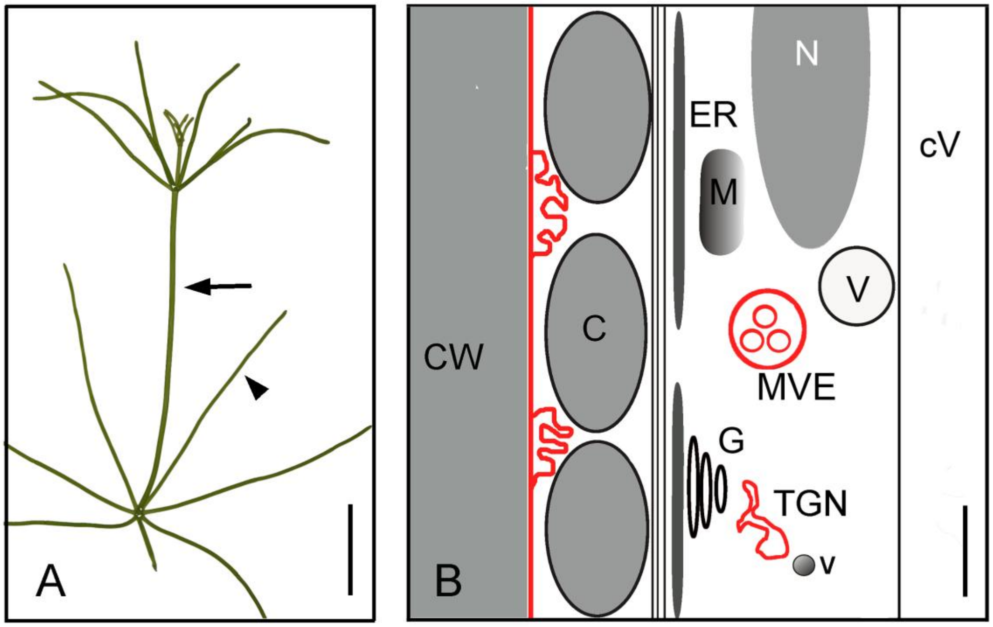

Fig. S 1. Characean thallus and schematic longitudinal section through internodal cell. (A) Thallus of *Chara australis*. Internodal cell of the main axis and of the branchlets are indicated by arrow and arrow head, respectively. (B) Schematic longitudinal section showing the cell wall (CW), the plasma membrane with smooth and convoluted regions (charasomes) and the stationary chloroplasts (C). Endoplasmic organelles like endoplasmic reticulum (ER), Golgi bodies (G), trans-Golgi network (TGN), vesicles (v), multivesicular endosomes (MVE), mitochondria (M), nuclei (N) and small vacuoles (V) move along actin filament bundles (parallel straight lines) attached to the inner chloroplast surface. The large central vacuole (cV) occupies more than 90% of the cell volume in mature internodal cells. Structures relevant in this study are shown in red. Bars are 1 cm (A) and 2 µm (B, only approximately drawn to scale).

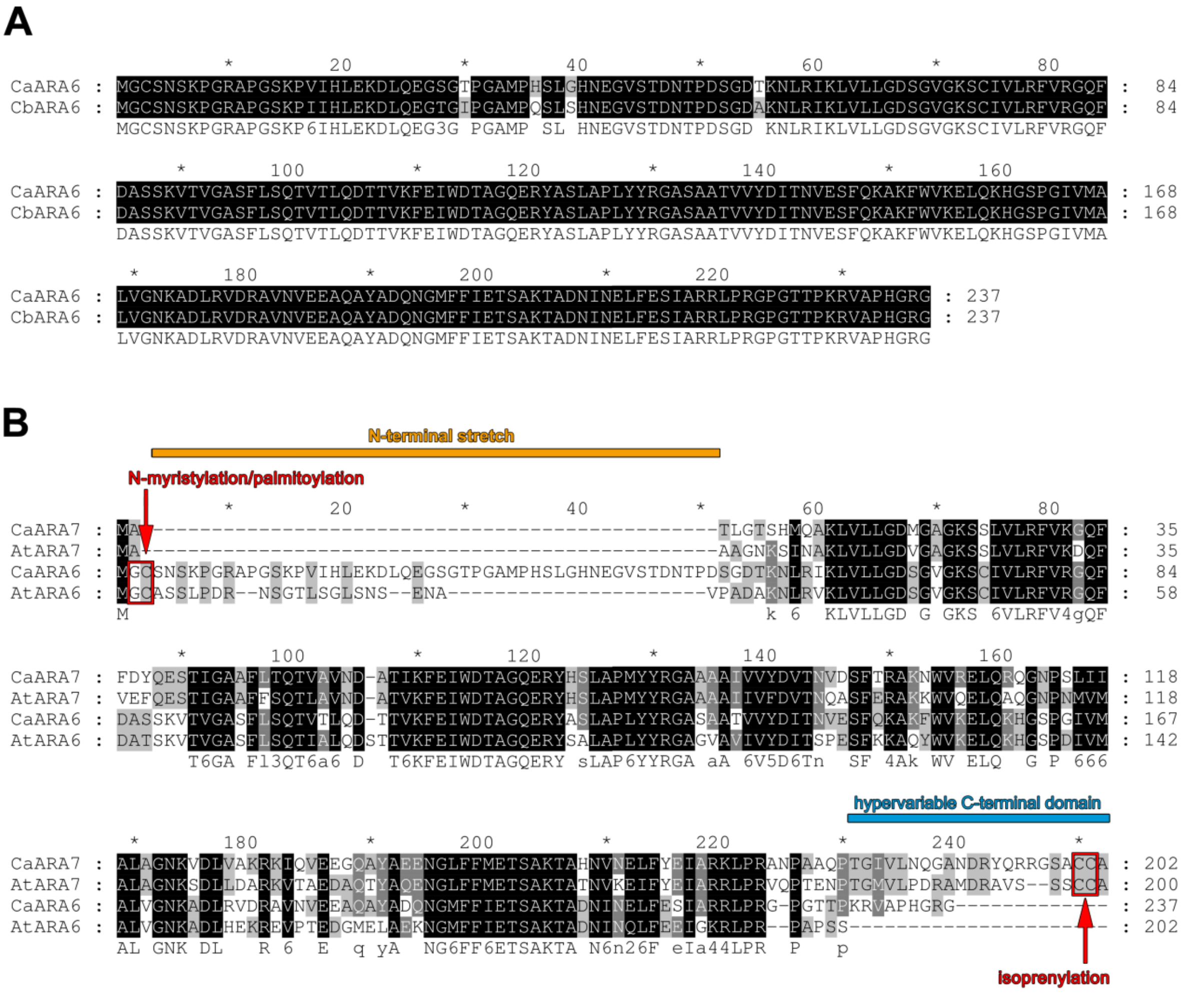

Fig. S 2. Sequence alignments of different ARA6 and ARA7 proteins. **(A)** ARA6 amino acid sequence alignment of *Chara braunii* (Cb) and *Chara australis* (Ca) proteins was performed using ClustalW. Identical residues are highlighted in black and conserved domains are displayed in different grey shades. **(B)** Multiple sequence alignment of amino acids from ARA6 and ARA7 proteins of *Arabidopsis thaliana* (At) and *Chara australis* (Ca). Amino acid residues numbering begins at the start methionine. Special characteristics of ARA6 proteins are N-myristoylation and palmitoylation sites, as well as the N-terminal stretch; ARA7 characteristics are the C-terminal hypervariable domain and the cysteine residues for isoprenylation.

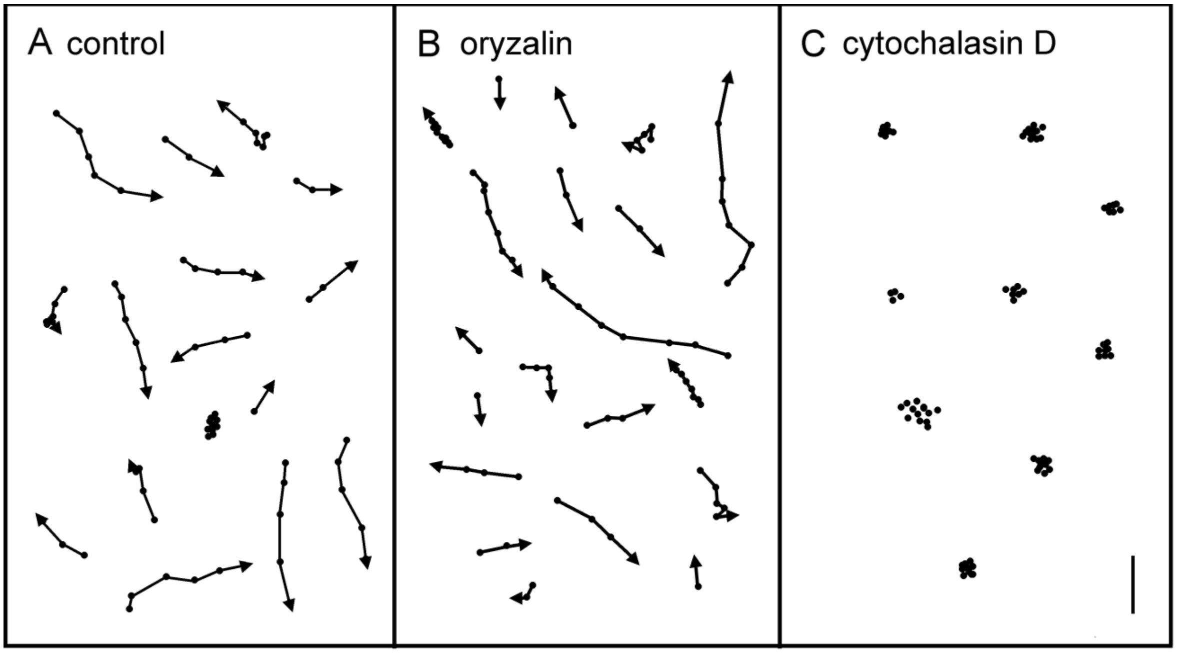

Fig. S 3. Actin-dependent dynamics of CaARA6-GFP transiently expressed in leaf epidermal cells of *Nicotiana benthamiana*. Trajectories of organelles in untreated control cells **(A)**, in cells treated with oryzalin to depolymerize microtubules **(B)** and in cytochalasin D-treated cells with a disturbed actin cytoskeleton **(C)**. Interval between positions (dots) is 660 ms; directions of movements are indicated by arrow heads in A and B. Bar is 5 µm.

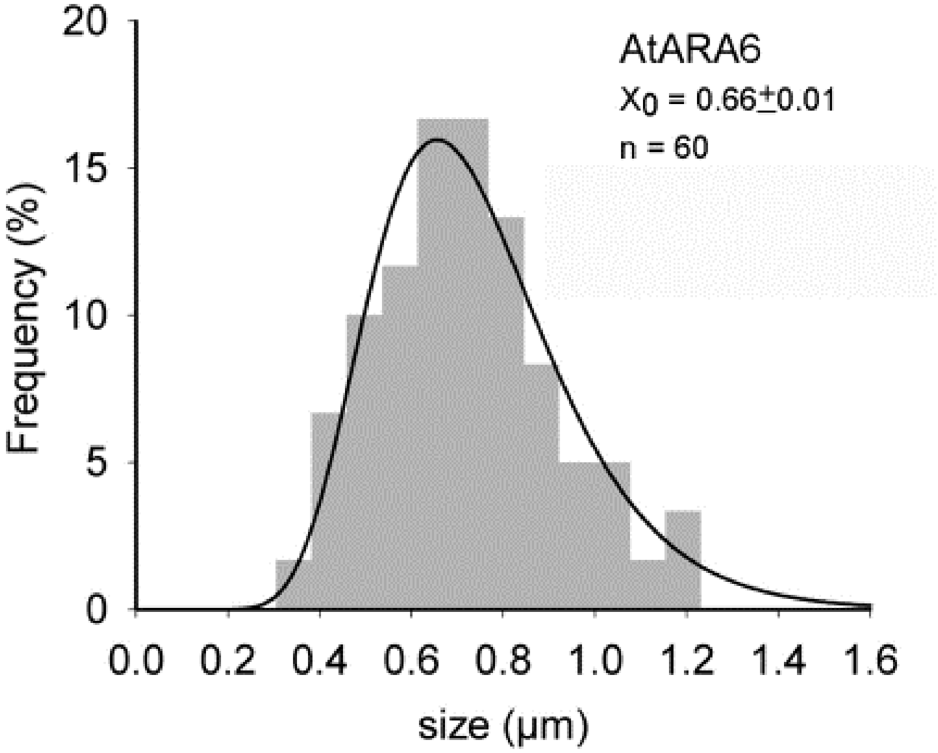

Fig. S 4. Size distribution plot of organelles labelled by AtARA6-GFP in transiently transformed leaf epidermal cells of *N. benthamiana.*

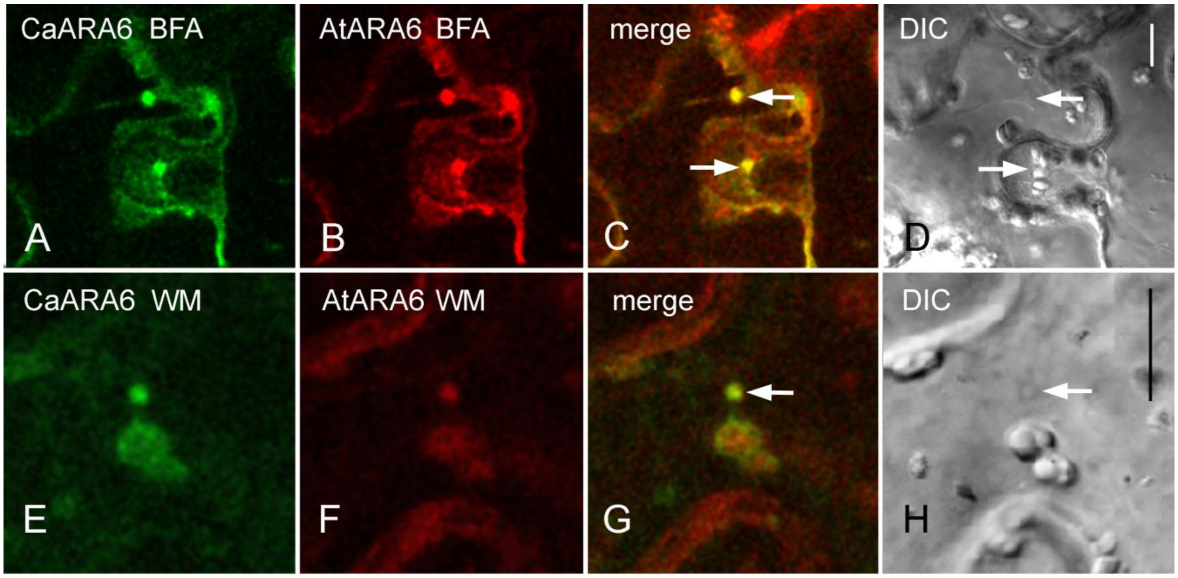

Fig. S 5. Tagged CaARA6 and AtARA6 co-localize at brefeldin A (BFA) and wortmannin (WM)-induced compartments when expressed in leaf epidermal cells of *N. benthamiana*. **(A-D)** BFA aggregates (arrows) labelled by CaARA6-GFP (A) and AtARA6-mCherry (B). C is the merged image, D is the DIC image. **(E-H)** WM compartment (arrow) labelled by CaARA6-GFP (E) and AtARA6-mCherry (F; G is the merged image, H is the DIC image). Bars are 10 µm.

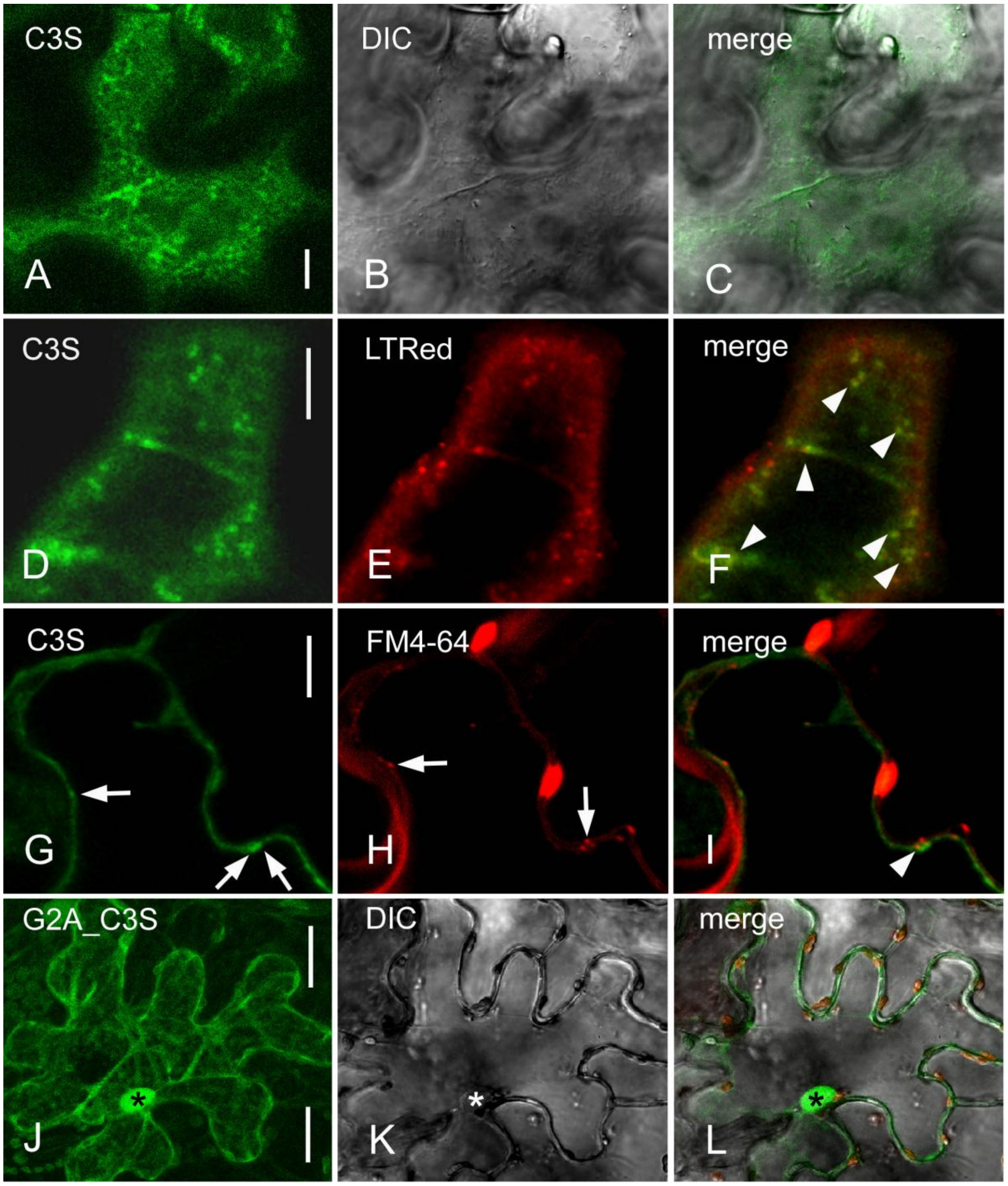

Fig. S 6. Leaf epidermal cells of *N. benthamiana* expressing GFP-tagged CaAra6^{C3S} (A-I) and CaAra6^{G2A_C3S} (J-L). **(A-I)** The mutant C3S which lacks the myristoylation site localizes to the cytosol (A-C) and to organelles stained by LysoTracker Red (LTRed; D-F) but rarely to FM4-64-labelled organelles (G-I). **(J-L)** The mutant G2A_C3S, which lacks the myristoylation and the palmytoylation sites is present in the cytosol and in the nucleus (asterisk). Arrows indicate organelles labelled either by GFP or by one dye; arrow heads in the merged images indicate organelles labelled by both markers. Bars are 20 µm (A-F) and 10 µm (G-L).

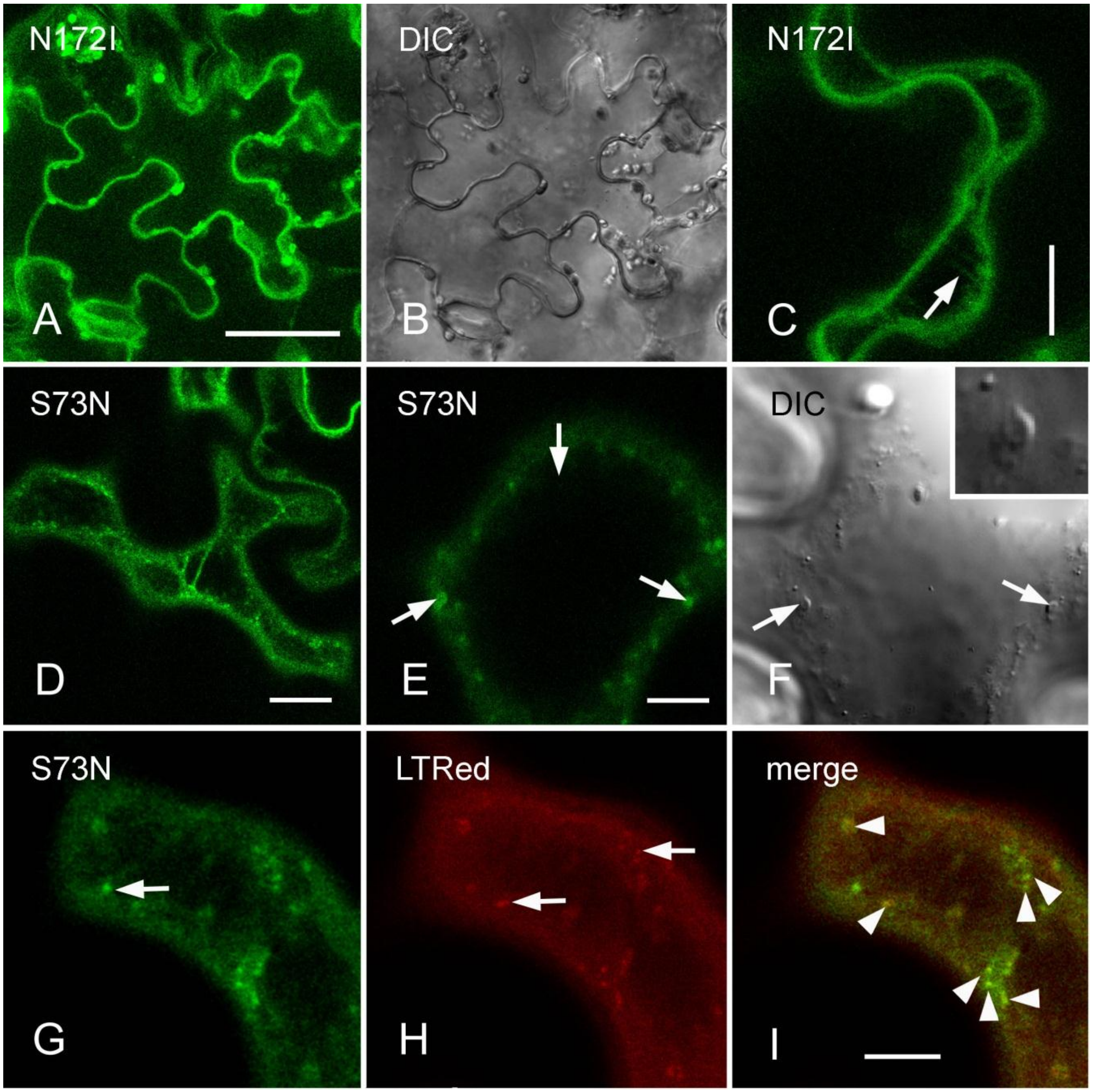

Fig. S 7. Leaf epidermal cells of *N. benthamiana* expressing GFP-tagged CaAra6^{N172I} (A-C) and CaAra6^{S73N} (D-I). **(A-C)** Nucleotide-free mutant CaAra6^{N172I} localizes to the cytosol and to the plasma membrane visualized by Hechtian strands in a cell plasmolysed in 300 mM sorbitol (C, arrow). **(D-I)** The GDP-bound mutant S73N localizes to the cytosol (D), to doughnut-shaped organelles (E and F, arrows; inset in F) and to particles which often co-localize with LysoTracker Red (LTRed; G-I). Arrows in G-I indicate organelles labelled by GFP or one dye only; arrow heads indicate organelles labelled by both markers. Bars are 50 µm (A-B), 10 µm (C, D-I) and 3.3 µm (inset in F).

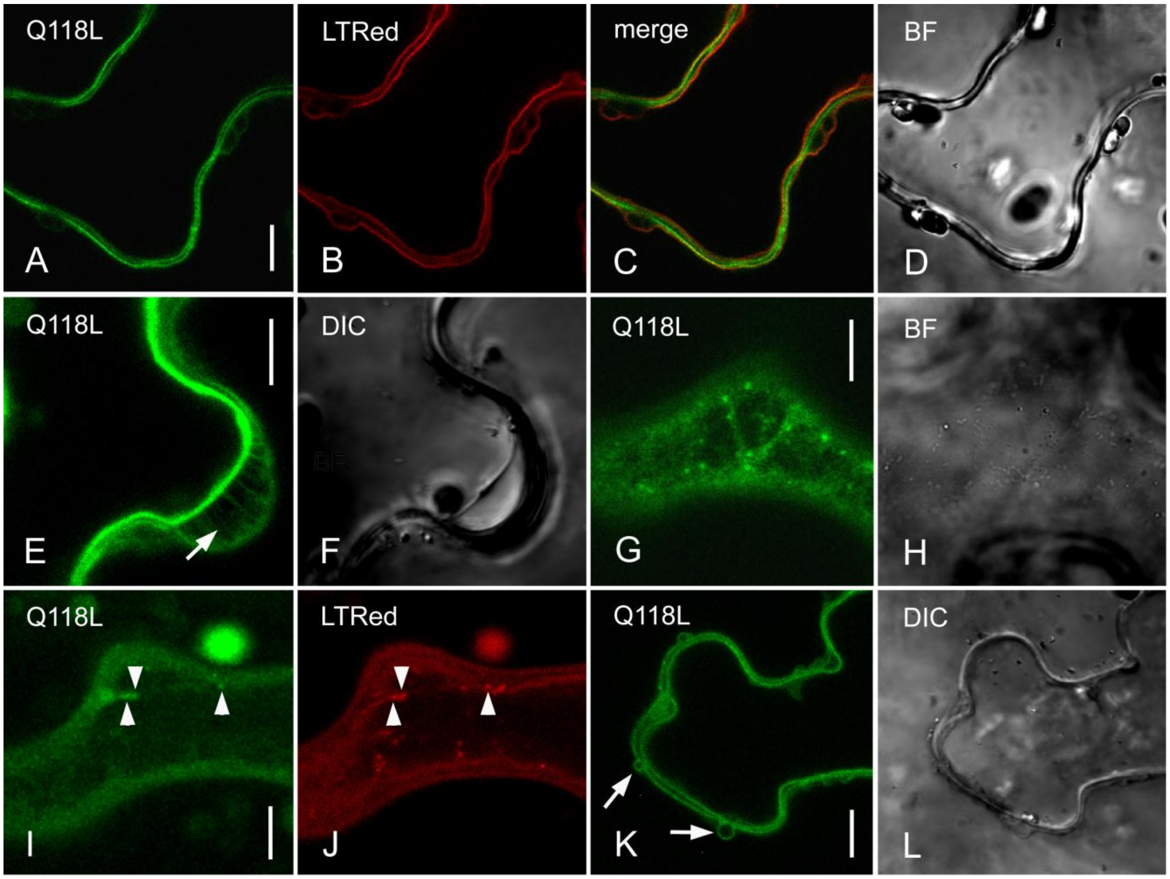

Fig. S 8. Leaf epidermal cells of *N. benthamiana* expressing GFP-tagged CaAra6^{Q118L}. **(A-D)** The GTP bound mutant localizes to the plasma membrane (A-D) of a cell in which the tonoplast was visualized by 3 hours incubation in 10 µM LysoTracker Red (LTRed). **(E-F)** In plasmolysed cells (300 mM sorbitol for 20 min) the Q118L mutant fusion localizes to Hechtian strands (arrow in E). **(G-L)** Occasionally, small organelles (G-J) or ring-like structures are labelled (arrows in K-L). The small organelles are also stained by LysoTracker Red (arrow heads in I and J). Bars are 10 µm.

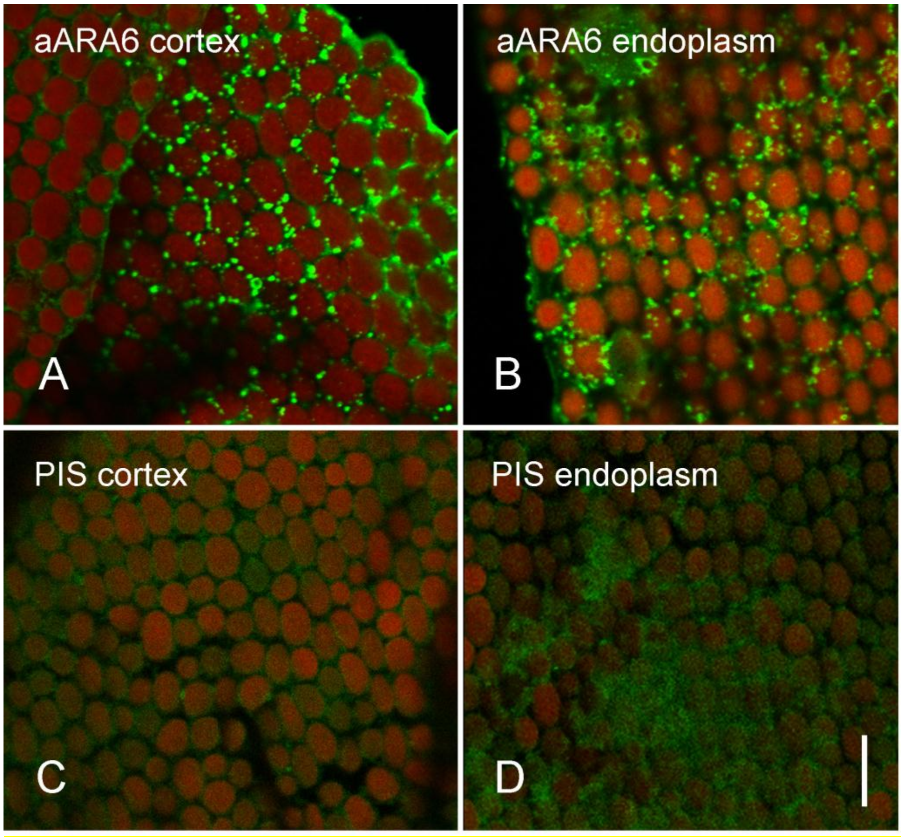

Fig. S 9. Immunofluorescence with a polyclonal antibody against AtARA6 (green) and autofluorescence of chloroplasts (red) in internodal cells of *Chara braunii*. **(A-B)** Immunofluorescence of charasomes (A) and of endoplasmic organelles (B). **(C-D)** Negative controls treated with rabbit pre-immune serum (C, cortex and D, endoplasm). Bar is 10 µm.

Video S 1. CaARA6-GFP-labelled organelles in untreated tobacco leaf epidermal cells. Time interval is 5 s.

Video S 2. CaARA6-GFP-labelled organelles in tobacco leaf epidermal cells infiltrated with 50 µM cytochalasin D for one hour. Time interval is 2 s.
